# Pleural Mesothelioma: Treatable Traits of a Heterogeneous Disease

**DOI:** 10.3390/cancers15245731

**Published:** 2023-12-06

**Authors:** Francesco Rocco Bertuccio, Francesco Agustoni, Giulia Galli, Chandra Bortolotto, Jessica Saddi, Guido Baietto, Nicola Baio, Simone Montini, Paola Putignano, Gioacchino D’Ambrosio, Angelo G. Corsico, Paolo Pedrazzoli, Giulia Maria Stella

**Affiliations:** 1 Department of Internal Medicine and Medical Therapeutics, University of Pavia Medical School, 27100 Pavia, Italy; francesco.bertuccio01@gmail.com (F.R.B.); f.agustoni@smatteo.pv.it (F.A.); gi.galli@smatteo.pv.it (G.G.); nicola.baio01@universitadipavia.it (N.B.); simone.montini01@universitadipavia.it (S.M.); paola.putignano01@universitadipavia.it (P.P.); corsico@smatteo.pv.it (A.G.C.); p.pedrazzoli@smatteo.pv.it (P.P.); 2 Cardiothoracic and Vascular Department, Unit of Respiratory Diseases, IRCCS Policlinico San Matteo, 27100 Pavia, Italy; 3 Department of Medical Oncology, Fondazione IRCCS Policlinico San Matteo, 27100 Pavia, Italy; 4 Diagnostic Imaging and Radiotherapy Unit, Department of Clinical, Surgical, Diagnostic, and Pediatric Sciences, University of Pavia, 27100 Pavia, Italy; c.bortolotto@smatteo.pv.it; 5 Radiology Institute, Fondazione IRCCS Policlinico San Matteo, 27100 Pavia, Italy; 6 Department of Oncology, Clinical-Surgical, Unit of Radiation Therapy, IRCCS Policlinico San Matteo Foundation, 27100 Pavia, Italy; j.saddi@campus.unimib.it; 7 Department of Radiation Oncology, Fondazione IRCCS Policlinico San Matteo, 27100 Pavia, Italy; 8 Cardiothoracic and Vascular Department, Unit of Thoracic Surgery, IRCCS Policlinico San Matteo, 27100 Pavia, Italy; guido.baietto@smatteo.pv.it; 9 Pathology Unit, Department of Diagnostical Services and Imaging, Fondazione IRCCS Policlinico San Matteo, 27100 Pavia, Italy; g.dambrosio@smatteo.pv.it

**Keywords:** pleural mesothelioma, treatable traits, novel therapeutic insight, CAR T cells therapy

## Abstract

**Simple Summary:**

Pleural mesothelioma is a neoplastic disease originating from mesothelium and is commonly associated with asbestos exposure. Despite the progress made, mortality is still high both because of late diagnosis and treatment resistance. With this review, we want to reassess all aspects of this disease from pathogenesis to diagnosis, and the most recent proposed treatment in order to inspire further research to cover unmet needs.

**Abstract:**

Pleural mesothelioma is an aggressive disease with diffuse nature, low median survival, and prolonged latency presenting difficulty in prognosis, diagnosis, and treatment. Here, we review all these aspects to underline the progress being made in its investigation and to emphasize how much work remains to be carried out to improve prognosis and treatment.

## 1. Introduction

Pleural mesothelioma (PM) is a particularly aggressive cancer arising from mesothelial cells lining the thoracic (pleura) cavity whose development has been related to the exposure to carcinogenic biopersistent mineral fibers, mainly asbestos [1]. The disease is characterized by heterogeneity, intended at several levels, which is ultimately responsible for the limited response to therapies and poor patient survival [2]. However, the advent of novel biotechnologies and next-generation approaches has also allowed for the identification of treatable traits with the development of targeted approaches for the otherwise neglected pathology. The aim of the work is thus—starting from a general description of the clinical–pathological features of the disease—to describe the known actionable targeted and the next-future drug pipelines.

## 2. Epidemiology

It is uncertain how common PM is worldwide. Driscoll et al. calculated that up to 43,000 persons per year die from the illness worldwide [3]. Although the use of asbestos is officially prohibited in 67 countries, Australia, Japan, North America, and western Europe together are thought to account for about 10,000 cases of mesothelioma each year [4]. With a median diagnostic age of 76 years, mesothelioma is a disease primarily affecting the elderly [5]. It is uncommon before the age of 50, but its frequency rises abruptly beyond that [5]. World-standardized incidence rates per 100,000 persons are 0.7 and 0.3 in the United States and 1.7 and 0.4 in Europe (for males and females, respectively). The Netherlands, the United Kingdom, and Australia are among the nations with the highest incidence of asbestos use in the past [6]. Along with relatively recent usage bans and a 40-year latency between exposure and presentation, incidence is still rising in many countries. Early in the new millennium, mesothelioma rates in Europe were rapidly increasing; nevertheless, due to the widespread home use of asbestos, the frequency of this disease is uncertain in the long run. Furthermore, the usage of asbestos is still growing in developing nations. Males are more likely than females to contract the disease, and females are claimed to have a higher rate of survival in several studies [7]. The World Health Organization (WHO) has identified PM as a very uncommon tumor that is directly linked to all forms of asbestos exposure, making it a preventable and industrial cancer. Compared to Europe, Central Asia still has a high rate of asbestos use, and some nations—including the US—only have usage limitations rather than outright bans [8].

## 3. Pathogenetic Basis

### 3.1. The Role of Asbestos

Due to its low cost-effectiveness and insulating qualities, asbestos has been widely used since the middle of the 20th century. Since the last decade of the 20th century, asbestos use has been strictly regulated in the United States and banned in Europe and Australia due to in vivo evidence of its carcinogenic potential [9]. The word “asbestos” is used in national regulatory papers to refer to six commercially exploited minerals: one serpentine (chrysotile) and five amphiboles (crocidolite, actinolite, tremolite, anthophyllite, and amosite). On the other hand, about 400 other minerals with comparable chemical and physical characteristics are found in the natural environment; their usage is uncontrolled, and they are not subject to regulations. Moreover, naturally occurring erionite fibers, which are even more carcinogenic than asbestos, have been utilized for building and road paving materials, and residents of some Cappadocian villages in Turkey and in some areas of North Dakota (US) are exposed to these fibers [10,11]. It is now commonly known that specific mineral fiber types have different carcinogenic potencies depending on their size, durability, dose, and physical characteristics [12,13,14]. Thin, long fibers have been linked to increased mutagenic and cytotoxic efficacy [15] due to the release of reactive oxygen species (ROS) and reactive nitrogen species (RNS) in response to “frustrated phagocytosis” [16,17,18]. However, due to its significantly lower inflammatory response when compared to carcinogenic fibers, palygorskite—a fiber that is highly prevalent in southern Nevada—was unable to promote cancer in vivo while having lower in vitro cytotoxicity and biopersistence [19]. The mechanisms underlying asbestos carcinogenesis have long been enigmatic [20,21]. However, mesothelial cells undergo an equivalent transformation under extended contact to chrysotile fibers [17,18]. Furthermore, it has been demonstrated that ROS released by asbestos-activated macrophages may indirectly cause DNA damage by forming 8-hydroxy-2′-deoxyguanosine (8-OHdG) adducts [22]. According to recent research on iron-catalyzed ROS production, asbestos-related carcinogenesis may entail ferroptosis, a non-apoptotic, iron-dependent cell death mechanism [23]. Furthermore, through stimulation of the PI3K/MEK5/Fra-1 axis, hepatocyte growth factor (HGF) has been implicated in asbestos-induced carcinogenesis [24]. However, human mesothelial cells (HM), a kind of cell that is especially vulnerable to fiber cytotoxicity and was previously thought to be apoptotic, die when exposed to asbestos particles [25]. Later, it was evident that tumor necrosis factor-alpha (TNF-α), an inflammatory mediator, was linked to the pathogenesis of asbestos [26]. One of the main contributing factors to the pathophysiology and carcinogenesis caused by asbestos and other mineral fibers known to cause cancer is chronic inflammation. The pro-inflammatory milieu created at the fiber deposition site by macrophages and PM, along with the biopersistence of many mineral fibers, enables the avoidance of cell death and ultimately leads to neoplastic transformation [27,28]. The passive release of high mobility group box 1 (HMGB1) by necrotic PM at the location of fiber deposition characterizes this controlled form of necrosis. One such damage-associated molecular protein (DAMP) is HMGB1, which encourages the macrophage recruitment necessary to maintain the chronic inflammatory process. To prime macrophages for inflammasome activation, HMGB1 binds to RAGE and other HMGB1 receptors. This, in conjunction with other stimuli, such as endogenous ROS produced following asbestos exposure, causes the NLRP3 inflammasome to assemble through the oligomerization of inactive NLRP3, apoptosis-associated speck-like protein (ASC), and procaspase-1. IL-1β, IL-18, IL-1α, and HMGB1 are released when the NLRP3 inflammasome is activated, initiating an autocrine chronic inflammatory process [29,30]. Additionally, TNF-α is secreted during this process, which stimulates NF-κB and increases HM’s chance of survival after asbestos exposure. Mesothelioma occurs because of the surviving HM’s continued proliferation and accumulation of genetic alterations. Furthermore, it was observed that ethyl pyruvate, which has been identified as an efficient HMGB1 inhibitor and suppressor of RAGE receptor expression, decreased the proliferation of mesothelioma cells both in vitro and in vivo. Both actions help to lower the malignancy of mesothelioma [31]. Whereas HMGB1 is primarily identified in the nucleus of HM, it has also been detected in the cytoplasm and nucleus of mesothelioma. HMGB1 is actively secreted into the extracellular space during mesothelioma, where it binds to RAGE and TLR receptors to form an autocrine pathway that stimulates the growth, motility, and survival of the cancerous cells [32].

### 3.2. Genetic Basis of the Disease

As not all PM patients have a history of asbestos exposure, asbestos fibers primarily cause mortality by necrosis and, to a lesser extent, through other cell death processes. Somatic gene mutations that impact DNA repair processes are frequently linked to carcinogenesis, as they cause an increase in the fraction of cells with damaged DNA and the accumulation of damage to DNA. Cancer may arise when these cells develop survival mechanisms like to those triggered by the HMGB1 pathway in mesothelioma. Inherited mutations that impact DNA repair and other genes may exacerbate the carcinogenesis process by making an individual more vulnerable to environmental carcinogens [33,34]. The present method used to investigate GxE interactions in the realm of carcinogens combines genetics (G) and environmental (E) investigations. Recently, the increase in the level of mutations in the genome of cancer cells has been linked to the catastrophic event known as chromothripsis. A single, segregated chromosome that is randomly reassembled can break, resulting in chromothripsis, which causes erroneous rearrangements or deletions of DNA sequences. As a result, huge genomic changes could happen after just one chromothripsis event. This elevated mutational status ultimately promotes carcinogenesis by favoring oncogene activations or the loss of tumor suppressor activities [35]. Notably, noncontiguous biallelic genome alterations with the characteristic pattern of chromothripsis and associated with possible neoantigen expression were found in genomic studies of mesothelioma cells and specimens. These findings may have intriguing implications for the immunogenicity of mesothelioma [36,37,38]. Mutations were discovered in several tumor suppressors connected to apoptosis and cell cycle regulation in human mesothelioma. The homozygous deletion on locus 9p21, which impacts the transcription of two tumor suppressors—p16INK4a and p14ARF—is one of the main genetic abnormalities seen in mesothelioma [39]. By attaching to CDK4 and CDK6, P16INK4a prevents cell proliferation, and p14 encourages apoptosis by preventing p53 ubiquitylation. Up to 80% of primary pleural mesotheliomas lacked p16, according to cytogenetic research, and p16 inactivation is associated with a worse prognosis [40]. Transgenic *p14* (+/−) mice showed decreased heterogeneity for p14 in their extracted primary mouse tumors, and these mice were more prone to asbestos-induced carcinogenesis [41]. Mesothelioma also exhibits significant mutations in Hippo signaling pathway intermediates. In almost 40% of cases of malignant mesothelioma, the upstream initiator of Hippo, neurofibromatosis type 2 (NF2)/Merlin, is inactive [42]. Remarkably, after BRCA1-related protein-1, NF2 is the second most often mutated gene in mesothelioma (*BAP1*). Compared to wildtype controls, heterozygous NF2 (+/−) mice showed an accelerated carcinogenesis and were more susceptible to asbestos exposure [43]. In the Hippo pathway, nonfunctional NF2 causes nuclear accumulation of WW Domain-contain transcription regulator (WWTR1 or TAZ) and yes-associated protein (YAP). One effect of the pro-inflammatory milieu created by asbestos fiber exposure is the increased nucleus formation of the YAP/TAZ complex, which, in turn, stimulates the expression of several proto-oncogenes and supports the survival of cancer cells [44]. It was feasible to identify potential frequent abnormalities at chromosome 3p21 in two unrelated US families with a high incidence of mesothelioma and no occupational asbestos exposure as a result of linkage analysis and array-comparative genomic hybridization (aCGH). Following sequencing, germline *BAP1* mutations linked to autosomal dominant transmission of uveal melanoma and mesothelioma were discovered [45,46]. The BAP1-related cancer syndrome was discovered in individuals with germline altered *BAP1* prone to additional cancer forms such renal cell carcinoma and squamous cell carcinoma [45,47,48]. According to recent research, BAP1 has multiple activities in the cytoplasm and nucleus that work together to prevent tumor growth. The endoplasmic reticulum (ER) fraction is the primary location of cytoplasmic BAP1, where it deubiquitylates and stabilizes the type 3 inositol-1,4,5-trisphosphate receptor (IP3R3). Via the mitochondrial uniporter channel (MUC) in the inner mitochondrial membrane and the voltage-dependent anion channels (VDACs) in the outer mitochondrial membrane, IP3R3 facilitates the release of Ca^2+^ from the ER into the mitochondrial space. The release of cytochrome c, which triggers apoptosis, is caused by an increase in Ca^2+^ concentration in the mitochondria. Reduced BAP1 dosage affects both DNA repair, accumulated DNA damage, and the apoptotic response in heterozygous BAP1+/− circumstances, such as in individuals in the families with the BAP1 cancer syndrome. This double function both favorably selects cells with cancer-causing mutations and encourages the growth of tumors [46]. The delineation of the intricate web of molecular processes mediated by asbestos carcinogenesis was aided by the discovery of BAP1 as a primary regulator of metabolism and cell death [48]. To fully understand the role of the genes predisposing to mesothelioma in the molecular pathways of asbestos carcinogenesis, more research will be necessary.

## 4. Diagnostic Work-Up

### 4.1. Clinical Presentation

The most common reason why patients with PM consult with a clinician is dyspnea, which is often accompanied by a dry cough, chest pain, exhaustion, and weight loss. Fever and sweating at night are less common symptoms. Patients with ascites as a secondary site of illness from pleural mesothelioma may exhibit early satiety and an unwillingness to lean forward (or in patients with peritoneal mesothelioma). The main reason for complaining about dyspnea is the increase in pleural effusion. When a physical examination suggests a pleural effusion, a first computed tomography (CT) scan and chest X-ray are requested. After draining the pleural effusion, the fluid is given a cytological analysis. Pleurodesis with talc poudrage is frequently carried out in the same surgical setting as pleural biopsy, which is frequently necessary for diagnosis. Early diagnosis depends on early detection and prompt study of the pleural or peritoneal effusion. Tumor growth will unavoidably result from a delayed diagnosis, which will reduce the available treatment alternatives. Due to increasing compression of the mediastinum and lung limitation, dyspnea and dry cough frequently persist after pleurodesis and get worse as the disease progresses. Weakening pain, loss of weight, and exhaustion are common indicators of mesothelioma progression. It is important to give these individuals the best dietary support possible. Pleural effusions should be drained whenever feasible to alleviate the symptoms. Often, distant metastases are absent or appear later. In a postmortem analysis of 318 patients with a pleural mesothelioma diagnosis, 55.4% of patients had distant metastases, and 53.3% had lymph node involvement. The spleen (10.8%), thyroid (6.9%), brain, and liver (31.9%) all showed signs of tumor spread (3.0%). Just 20% of patients had their exact cause of death determined; pulmonary emboli and bronchopneumonia were the most common causes. Additional reasons included invasion of the major vessels and cardiac tamponade. Up to 25% of patients had cachexia, which was more common in cases when there was no apparent reason for death [20,49].

### 4.2. Imaging

Due to its widespread availability and relatively low cost, CT is currently employed for the diagnosis, staging, and post-treatment monitoring of malignant pleural lesions. CT can identify the thickness of the pleura, indicate the existence of malignant characteristics, and help ensure that biopsies are performed correctly. Several authors have demonstrated the usefulness of CT in separating benign from malignant pleural illness. Nodular pleural thickening, mediastinal pleural thickening, parietal pleural thickening > 1 cm, and circumferential pleural thickening are useful characteristics for distinguishing malignant illness on CT scanning. The specificity of these findings is noticeably lower, even though the sensitivity is higher than previously reported (68%), according to data from a recent study (78%). Notably, malignant pleural illness is not ruled out in the absence of these results, with a negative predictive value of 65% [50,51]. CT can be helpful for the assessment of intrathoracic lymphadenopathy.

Since magnetic resonance imaging (MR) has shown to provide a superior soft tissue contrast than computed tomography (CT), it is occasionally utilized for staging. MR is more sensitive in displaying invasion of the diaphragm and chest wall, but it is less sensitive in detecting lymph node metastases and visceral pleural invasion. When iodinated contrast medium is contraindicated, magnetic resonance imaging (MR) is utilized to obtain a more precise evaluation of chest wall or diaphragmatic invasion in patients who may be candidates for aggressive multimodality therapy regimens [50]. MR is not frequently utilized in the diagnostic and staging evaluation of PM patients due to cost concerns, limited availability, and lengthy imaging times. However, because of its superior contrast resolution on unenhanced scans and better post-contrast enhancement, it has been proven to be helpful in instances that are unclear or in patients who may benefit from multimodality therapy, including surgery. When compared to muscle, PM is characterized by an intermediate to slightly hyperintense signal on T1-weighted sequences and a more hyperintense signal on T2-weighted sequences. For identifying interlobar fissure invasion and neighboring structure invasion, the most sensitive sequences are contrast-enhanced T1-weighted fat suppressed sequences. Moreover, diffusion-weighted magnetic resonance imaging can disclose details about tissues by measuring the diffusivity of water molecules in certain tissues. Using this method, signal loss may be quantitatively evaluated using the apparent diffusion coefficient (ADC), which is based on the diffusion of water molecules being restricted by macromolecules and cell membranes. This method indirectly provides tissue cellularity information. In the diaphragm and chest wall, Patz et al. demonstrated that MRI was marginally more sensitive than CT for predicting resectability (100% vs. 93–94%, respectively). This is probably because MR offered superior soft tissue contrast in those areas [52]. Heelan and colleagues examined the preoperative staging accuracy of 65 MPM patients using MR versus CT. While the diagnostic accuracy of MR and CT imaging was about equal when it came to staging, MR imaging was superior when it came to identifying isolated foci of invasion of the chest wall, endothoracic fascia involvement, and diaphragmatic invasion assessment. These results, however, did not alter the surgical strategy [53]. Additionally, multidetector CT (MDCT) offers greater resolution and multiplanar reformations, which may lead to a more accurate assessment of the disease’s local extent [50,51].

Furthermore, PET-CT, even though it is not specific enough to routinely identify PM, can be utilized to provide helpful functional information on pleural lesions in cases when prior talc pleurodesis has not been carried out. Indeed, PET-CT during the initial period post talc pleurodesis will enhance due to the inflammatory process of the pleurodesis, but later on (i.e., after 3 months), it can be a useful tool for assessing the extent of the disease and the response to treatment [54]. Based on the standardized uptake value (SUV), PET has shown to be quite useful in differentiating between benign and malignant disorders. It also helps with decision making on staging and possible treatment options. Several authors demonstrated that malignant may be distinguished from benign pleural illness with sensitivities of 91% to 100% and specificities of 78% to 100% using an SUV cutoff value of 2.0 to 2.2 [55]. Furthermore, it has been discovered that PET-CT is helpful in determining the best biopsy site to obtain a definitive diagnosis. However, false-negative and false-positive results in pleural disease limit the accuracy of PET-CT (concomitant asbestos-related disease, parapneumonic effusion, and uremic pleural disease). PET-CT has proven to be more accurate in both determining prospective candidates for multimodality therapy, which includes invasive surgical procedures, and in staging MPM patients overall. Frauenfelder et al. recently assessed the precision of PET-CT and CT for PM staging in 28 patients receiving induction chemotherapy [56]. When it came to preoperative staging in the International Mesothelioma Interest Group staging system (10), PET-CT had a greater accuracy rate than CT (91% vs. 82%, respectively). Additionally, PET-CT may be useful in tracking the effectiveness of treatment, identifying recurrent illness, and supplying prognostic data for PM patients [57,58].

When it comes to thoracentesis and drain placement—the primary palliative care intervention for cases of advanced PM—ultrasound (US) plays a crucial role in directing the needle placement process. US has the obvious benefits of being quick, easy, and affordable, but it also has the drawback of being very user dependent [51]. There is a greater than 95% specificity for malignancy in pleural-based mass lesions, pleural thickening greater than 1 cm, nodular pleural thickening, and diaphragmatic nodularity [59]. Qureshi and colleagues showed that US can distinguish between malignant and benign effusions with an overall sensitivity of 79% and specificity of 100%, with specificity comparing favorably with CT, by using similar morphologic criteria as those used in CT (pleural thickening > 1 cm, pleural and diaphragmatic thickening > 7 mm) [60].

### 4.3. Bioptic Procedures

**Figure 1:**
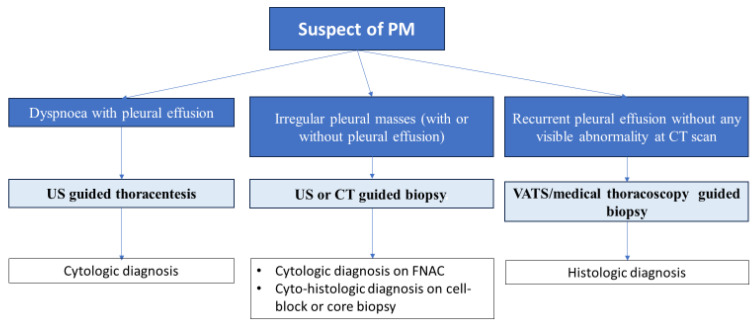
Diagnosis of PM. It should always be based on the results obtained from an adequate biopsy (less commonly, an exfoliative or fine-needle aspiration cytology evaluation) in the context of appropriate clinical, radiologic, and surgical findings. Effusion cytology for MPM definitive diagnosis remains controversial and biopsy is recommended especially for histological subtyping. VATS allows for the exploration of entire pleural surface and enables targeted biopsies, providing material samples for both histological examination and immunohistochemical analysis. A history of asbestos exposure should not be taken into consideration by the pathologist when confirming or excluding PM [5,51,65].

Pathological samples are routinely collected in response to suspected disease presentations (Figure 1). It should be mentioned that IHC stain can be applied to the material (including cell blocks) in the event of effusion fluid sampling or percutaneous fine needle aspiration cytology of a region of pleural thickening. Indeed, it is essential to underline that cytology is negative in about 50% of the cases [61]. Therefore, video-assisted thoracic surgery (VATS) is necessary to confirm or exclude diagnosis. A minimum of three representative biopsies from different sides are needed to determine histologic tumor subtype. Under local or general anesthesia, thoracoscopic biopsies are considered the gold standard when examining an unexplained pleural effusion in cases where mesothelioma is a possible differential diagnosis. Image-guided cutting-needle biopsies, on the other hand, could be of help in those limited cases who cannot undergo safe awake thoracoscopy/pleuroscopy under local anesthesia and sedation. For pleural cancer, medical thoracoscopy has a high diagnostic yield. The review of 47 trials involving 4756 individuals revealed extremely low complication rates: 0.34% had serious difficulties, 1.8% had major complications, and 7.8% of cases had moderate complications. Pleural biopsies have a 94% negative predictive value, 100% specificity, and 95% sensitivity. The added advantage of VATS is that it permits more invasive surgical procedures to be carried out concurrently with the diagnostic process, such as tumor debulking and lung resection. It is noteworthy that individuals who are not intubated can undergo VATS under local anesthesia. To obtain a histological diagnosis, medical thoracoscopy is a safe procedure that involves forceps biopsy sampling from at least three distant sites to determine histologic subtype. Additionally, the apparently normal pleura may be sampled in a representative manner, and areas of interest may be targeted using thoracoscopic imaging [62,63,64]. Parietal pleural biopsies need to be sufficiently deep to assess the invasion of the chest wall’s muscle and fat [54]. VATS may not always be possible because of an obliterated pleural space (e.g., low and loculated effusion) caused by a locally advanced disease. In these situations, an open pleural biopsy can be performed through a tiny, muscle-sparing incision made inside the intercostal space; alternatively, via cutting-needle biopsy guided by CT or TUS. Thoracotomy is therefore typically not required for a precise diagnosis of PM [62,63,64].

### 4.4. Pathologic Classification and Staging

Pleural tumors have been updated in the WHO Classification of Thoracic Tumors, Fifth Edition, which was published in 2021. While the three primary histologic subtypes—epithelioid, biphasic, and sarcomatoid—remain, the classification of mesothelial tumors has undergone substantial modifications since 2015. All mesotheliomas are considered to be malignant; so, the term “malignant” has been dropped from the descriptions of diffuse and localized mesothelioma. Because localized mesotheliomas have been linked to a better prognosis when totally removed, they continue to be distinguished from diffuse mesothelioma. Recent developments in the understanding of mesothelioma genomes have resulted in a greater awareness of mesothelioma in situ (MIS), a previously unrecognized condition. Criteria for MIS have now been established and are included in the 2021 WHO classification [66,67].

**Figure 2:**
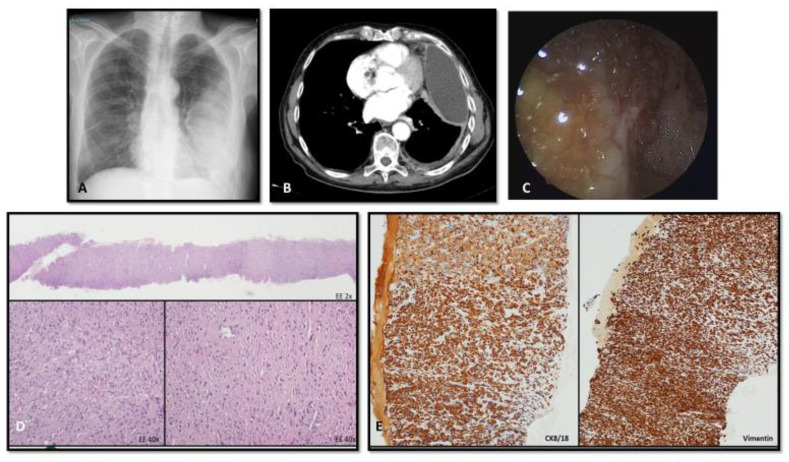
ICH profile is required to detect the primary origin of a pleuric lesion. Imaging (**A**,**B**) and thoracoscopic (**C**) view of a pleuric malignant lesion. Cytologic analysis on needle biopsy sample showed site of diffuse infiltration of poorly differentiated neoplasm (**D**), immunoreactive for vimentin 8 (**E**), and negative for TTF1, p40, calretinin, WT-1, D240, cytokeratin 5/6, vimentin, S100, BER-EP4, CEA, CD31, CD34, desmin, myogenin. In this case, the IHC stain profile is not able to solve the tissue/lineage of origin of the lesion and differential diagnosis encompassed undifferentiated PM, small/dormant lung primary carcinoma, pleural localization of melanoma, of sarcoma, ectopic lung epithelial cells which undergo malignant transformation and pleural localization of epithelial cancer from unknown primary site of origin.

Immunohistochemistry (IHC) is advised because of the variety of histological abnormalities seen in mesotheliomas, the pleura’s frequent location for metastatic illness, and reactive alterations that exhibit considerable atypia. These factors make morphology-only diagnosis of mesotheliomas hard. A combination of two “mesothelioma-associated” markers (e.g., calretinin, Wilms’ tumor-1 (WT-1), cytokeratin 5/6) and two “(adeno)carcinoma-associated” markers (e.g., CEA, Ber-EP4, MOC-31) can typically be used to diagnose epithelioid mesotheliomas. Additional markers may be added based on the possibility of known, suspected, or occult malignancies (Figure 2). While most sarcomas are negative and most sarcomatoid mesotheliomas are positive for broad-spectrum cytokeratins, these other markers are far less sensitive and specific in sarcomatoid malignancies. It is also important to separate diffuse and localized mesotheliomas from adenomatoid tumors and well-differentiated papillary mesothelial tumors, which are both of mesothelial origin but exhibit significantly more passive behavior. Pleural effusion cytology, blind or image-guided needle core biopsies, open or VATS surgical biopsies (Figure 2), macroscopic complete resection (MCR), extended pleural decortication (EPD), and extrapleural pneumonectomy (EPP) samples are among the samples used to diagnose pleural mesothelioma. Though opinions on cytological diagnosis accuracy vary, most will require at least IHC on cell blocks to confirm mesothelial phenotype. Tissue biopsies that enable the determination of subpleural invasion and its magnitude are usually necessary. Recent research has revealed that mesotheliomas frequently exhibit loss of BAP1, which is more common in the epithelioid subtype, and/or loss of CDKN2A, which is relevant to the differential diagnosis of the epithelioid subtype versus mesothelial hyperplasia and sarcomatoid subtype versus reactive fibrous pleuritis (more common in sarcomatoid subtype). IHC can be used to detect BAP1 loss. While loss of methylthioadenosine phosphorylase (MTAP) staining can serve as a surrogate (with 96% specificity but 78% sensitivity) in the event of CDKN2A loss, molecular analysis is necessary for MTAP loss. This is because MTAP is located at 9p21.3, extremely close to CDKN2A. However, it is advised that any labs utilizing these antibodies or molecular assays have testing procedures that have been verified. Since the science is still very young, molecular testing should not be used in isolation from other findings [5].

The PM staging system has undergone modifications with the release of the eighth version of the TNM classification. The most significant change to the T-component is the combination of stages T1a and T1b into a single category, T1. Thus, the difference between malignancies confined solely to the parietal pleura and tumors infiltrating both the parietal and visceral pleura is no longer valid. This was a result of the prognosis’s lack of significance [63]. Since both intrapleural and extrapleural nodes are now included in category N1, the revised TNM now only has two N categories (N1 and N2). Moreover, N2 disease has replaced the earlier anatomical characteristics for N3 disease. The number of nodes involved appears to have a greater impact on survival than the precise anatomical locations of nodal illness [64]. It is well established that individuals with PM who develop nodal metastases have far poorer survival rates than those who do not [68,69]. The node categorization was split into four primary groups under the former TNM staging system: N0, N1, N2, and N3. The numbers N0, N1, N2, and N3 denote the presence of nodal metastases to the ipsilateral bronchopulmonary, hilar, or internal mammary nodes, subcarinal, ipsilateral internal mammary, or mediastinal nodes, and all contralateral intrathoracic and supraclavicular nodes. On the other hand, a change was suggested regarding the anatomical positions of the lymph nodes and the classifications that correspond to them. The eighth edition of the staging system, which is new, groups intrapleural (prior N1) and extrapleural (previous N2) lymph nodes into one category (N1) and moves previous N3 nodes into N2 category [70].

## 5. Treatment

### 5.1. The Role of Surgery and Radiotherapy: An Update

**Figure 3:**
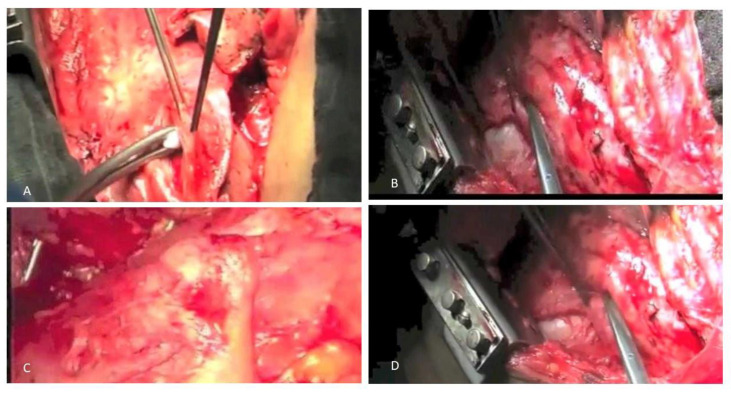
Surgical approach to PM: (**A**,**B**,**D**) lung decortication; (**C**) extrapleural pleurectomy.

The next crucial phase is management, which comes once the diagnosis has been verified. Regretfully, there are not many proven treatment options for PM. However, in the early stages of the disease, patients with high-performance status may be able to access multimodal therapeutic techniques, which include radiation therapy, chemotherapy, and surgery (trimodal approach). It should be underlined that trimodal approach is not the only option and that pleurectomy/decortication with adjuvant chemo has much lower morbidity and better overall survival (Figure 3).

In certain cases, where a full macroscopic resection is anticipated, surgery may be recommended [70,71]. For the reasons listed below, surgical management of pleural mesothelioma is still debatable. Aggressive surgical therapies have not been shown to improve survival, and there are numerous unmet needs and divergent viewpoints in this regard. The T and N categories, the patient’s performance status, and their cardiopulmonary reserve all play a role in the selection of patients for surgical treatments. EBUS, mediastinoscopy, and clinical staging techniques, however, have poor correlations with pathological staging. With the goal of curing, two procedures are carried out: pleurectomy/decortication (P/D) and extrapleural pneumonectomy (EPP).

The severity of the illness and the patient’s physiological reserve, notably their ability to breathe on their own, have an impact on surgery and the kind of procedure that is carried out. The goal of both P/D and EPP is to accomplish a macroscopic full resection of every tumor. EPP entails removing the pleura and the lung beneath it, typically including the diaphragm and pericardium.

P/D entails total pleural excision without lung underneath. Known as an extended P/D, or EPD, this procedure includes pericardial and diaphragm excision as well [72,73]. Nonetheless, over the last ten years, several studies have demonstrated that OS following EPP is most likely lower than OS following P/D or EPD, and that the morbidity and mortality of EPP is higher (mortality ranging from 6% for EPP to 3% for P/D or EPD) [74]. Only a very small subset of younger patients with an epithelioid mesothelioma histological subtype and no lymph node metastases may benefit from enhanced long-term OS with EPP, according to data from the IASLC database [75].

The body of research supporting aggressive surgical resections, such EPP or EPD, primarily draws from single-institution series of highly chosen individuals with early-stage, low-disease-burden epithelioid histology mesothelioma. While patients who are expected to have longer survival times are selected based on baseline clinical characteristics, the median survival following a major debulking surgery is commonly reported as 14–18 months following P/D or EPP, which is essentially the same as among patients who do not undergo surgery [76,77].

In 2011, the MARS study was published; the aim of authors was to assess effects of extra-pleural pneumonectomy (EPP) on survival and quality of life in patients with malignant pleural mesothelioma [78]. These data, although limited, suggested that EPP within trimodal therapy offers no benefit and possibly harms patients [71,79].

Moreover, in October 2023, researchers presented highly anticipated results from the MARS2 clinical trial; the study compared survival for two groups of pleural mesothelioma patients: one group had chemotherapy with surgery (extended pleurectomy decortication), and the other had chemotherapy without surgery. The results showed no significant difference in the survival time between the two groups [70].

Multimodality therapy is often used for clinical stage I to III pleural mesothelioma. However, the optimal combination therapy remains debated. In this context, the sequential combination between surgery and chemotherapy also deserves better investigation. Preliminary results for a case series of our group have shown better short-term outcomes with upfront extended pleurectomy/decortication (EDP), followed by adjuvant chemotherapy (CT) if compared to EDP preceded by neoadjuvant CT [80].

Surgery for Mesothelioma After Radiation Therapy (SMART) entails administering an accelerated, hypofractionated hemithoracic regimen that delivers 25 grays (Gy) in five fractions along with a boost of 5 Gy to the gross disease after an induction dose of hemithoracic radiation is administered prior to surgery. The overall median survival for epithelioid mesothelioma patients treated with this SMART strategy was 36 months, which is encouraging [81,82]. As a component of multimodality therapy, immunotherapy may be introduced on the best possible platform thanks to the SMART method. Other methods have also been investigated in the multimodality context besides radiation. Tumor resection (P/D or EPP) plus intraoperative lavage with chemotherapeutic chemicals is the most used combination. Different drugs such as cisplatin, doxorubicin, mitomycin C, and gemcitabine have been used for this procedure [19].

Despite the identification of long-term survival, this treatment is still deemed experimental. Success with photodynamic treatment has been patchy [83,84]. Using this method, a photosensitizing drug is first administered to the thoracic cavity, and then laser light is applied, causing a cell death that penetrates the postsurgical tumor bed up to a few millimeters. An alternative strategy that involves using a gel containing cisplatin following resection is presently undergoing evaluation. Due to the severity of their condition, age, comorbidities, or low performance status, the majority of patients are not recommended for surgery and are instead given consideration for palliative chemotherapy.

With US Food and Drug Administration (FDA) approval in 2004, the gold standard of medical treatment for mesothelioma has been the combination of cisplatin and pemetrexed [20,85]. This cytotoxic regimen is routinely used either before or after surgery. A recent clinical trial demonstrated that the addition of bevacizumab improves survival compared to the use of the platinum-doublet alone, although this regimen has not been approved by the FDA to date [21,86,87]. However, the median survival for resectable pleural mesothelioma remains at 17 to 25 months and, for unresectable mesothelioma, it is 9 to 12 months [22]. In the end, palliative procedures such as partial pleurectomy or VATS with pleurodesis could be performed to treat recurring pleural effusions or to re-expand a partially entrapped lung. The role of radiotherapy is limited; it can be considered a palliative treatment in the case of mesothelioma-induced pain. Cho et al. developed a protocol that starts with hemithoracic radiation to deliver an optimal dose of radiation to the tumor before surgical resection [18].

### 5.2. Targeting PM Immune Landscape: Current Strategies

Using RNA sequencing, Bueno et al. found four distinct molecular expression phenotypic groups in 212 mesothelioma patients, each with varying associated characteristics related to survival and mutation. A lower chance of survival was linked to the expression of programmed death-ligand 1 (PD-L1), which was present in 39% of patients. There was an increase in PD-L1 expression in nonepithelial mesotheliomas [88]. Cytotoxic T-lymphocyte-associated protein 4 (CTLA-4) inhibitors were not able to increase mesothelioma patients’ survival in clinical trials [89].

Trials after this one revealed that PD-L1 inhibitors might help certain patients [90,91]. There are currently several mesothelioma checkpoint inhibitor trials underway. These include antibody-drug conjugates, chemotherapy, and PD-L1 inhibitor combos with CTLA-4 inhibitors. The DREAM study, which examined the addition of the PD-L1 inhibitor durvalumab to standard-of-care chemotherapy (cisplatin and pemetrexed, up to 6 cycles), examined the use of durvalumab in the first-line metastatic context. Maintenance durvalumab was administered every three weeks [92]. With a median progression-free survival of 6.9 months, the primary goal of progression-free survival was 57% at 6 months. The response time was 6.5 months on average.

It was deemed acceptable to tolerate the extra toxicity, which included three patients who required corticosteroid therapy due to grade 3 autoimmune toxicity. Furthermore, the 2018-started CheckMate743 study (NCT02899299) examined the potential advantages of combination immunotherapy (nivolumab plus ipilimumab) over conventional chemotherapy [93]. 

The study was positive, showing a survival benefit for the immunotherapy arm. The larger benefit emerged for the sarcomatoid mesothelioma histotype. As a consequence of this study, first-line nivolumab plus ipilimumab has been granted FDA approval for the treatment of advanced mesothelioma [23]. In second line, the trial CONFIRM documented a survival benefit with nivolumab in comparison with placebo [24,94]. Reported toxicities across trials are comparable to the use of immunotherapy in other tumors and can be managed with the standard of care [25,26,27,28,95]. A priority is the identification of biomarkers that predict benefit or harm from immune checkpoint inhibitors.

The phase III DREAM3R trial is presently looking at it after combined chemotherapy with durvalumab as a first-line treatment showed encouraging results. In addition, the phase III BEAT-MESO trial is still in progress, comparing bevacizumab to conventional chemotherapy with or without atezolizumab [92,96]. A further tactic to combat immunological suppression in mesothelioma takes into account dendritic cells’ capacity to stimulate T-cells. When presented to T-cells, tumor antigens can be recognized by dendritic cells.

Dendritic cell vaccine therapy has been used in a number of phase I/II trials. These distinct phase I/II trials’ long-term follow-up revealed a positive signal, with a 2-year OS of >50% and a 5-year OS of >20 percent. The current randomized phase III DENIM trial is a result of these studies; the accrual has been finished and the results are pending [97,98]. The enzyme pegargiminase (ADI-PEG 20) breaks down arginine, an amino acid that is essential to mesothelioma cells. This medication is being studied in combination to first-line chemotherapy in the ATOMIC phase II/III trial, in comparison to a placebo [99].

### 5.3. Cell Therapy Approaches

#### 5.3.1. CAR T-Cell Therapy

An alternate strategy to deal with the problem of inactivated T-cells is CAR T-cell therapy. This approach involves administering genetically modified T-cells against a particular tumor-associated antigen, like mesothelin. Numerous phase I studies have investigated this approach, mostly in combo therapies like immune checkpoint inhibitors [100,101]. Immunotherapies using CAR-T cells are a major development in the treatment of blood cancers, and great efforts are being made to successfully apply them in the battle against solid tumors. Tumor-associated antigens’ (TAAs) selection is important. Solid tumors frequently lack distinct TAA expression, and while MHC antigen recognition does not restrict CAR-T cell activity, TAA expression on the tumor cell surface is still necessary for CAR-T cell activity. Therefore, the ideal choice for a therapeutic target is to choose a TAA that is overexpressed by tumor cells while maintaining as much physiological expression as feasible. Such a possible antigen is mesothelin (MSLN), which is normally expressed largely on the mesothelial surfaces of the pleura, peritoneum, pericardium, and the tunica vaginalis (in males), but is markedly overexpressed in a wide variety of solid tumors. Numerous preclinical models and clinical trials have assessed anti-MSLN CAR-T cells. Other tumor-killing strategies, such as antibody-based medications and anti-cancer vaccinations that target MSLN, have also been developed and evaluated, both in experimental and clinical settings, in addition to anti-MSLN CAR-T cells. Fewer clinical trials are now being conducted for anti-MSLN vaccinations due to their mild and generally less beneficial effects in the clinic when compared to CAR-T cells and anti-MSLN antibody-based therapies. They can also secrete pro-inflammatory substances that draw in and activate other immune cells within the tumor microenvironment. In order to improve their safety profile, if necessary, anti-MSLN CAR-T cell engineering with suicide genes like iCasp9 could mitigate the negative effects of excessive CAR-T cell activation, such as CRS [102]. Using humanized or fully human anti-MSLN scFv is a potential tactic that may improve the survival of CAR-T cells and lessen the possibility of immunogenic reactions. Comparably, medications based on humanized or fully human anti-MSLN antibodies, such as amatuximab and anetumab ravtansine, have better safety profiles, a longer half-life, and a lower immunogenicity [103,104,105,106]. Numerous phase I/II clinical trials using amatuximab (and anetumab rav-tansine) to treat patients with solid tumors expressing MSLN have either concluded or are ongoing.

On the other hand, it has been demonstrated that antibody-based medications like SS1P, which has a murine anti-MSLN moiety, are extremely immunogenic and cause patients to produce neutralizing antibodies against it [106]. As a result, LMB-100/RG7787, a less immunogenic bacterial exotoxin, and a humanized anti-MLSN antibody have proven to be a more effective immunotoxin than SS1P [106,107]. Anti-MSLN antibody-based medications and CAR-T cells have had a difficult time efficiently entering solid tumor locations. Intravenous delivery of antibodies and CAR-T cells is still widespread, and frequently results in both inadequate anti-tumor effectiveness and toxicity that is “on-target/off-tumor” [102].

#### 5.3.2. STING

Immunological weariness and immune exclusion are two obstacles to the immunotherapy response that can be addressed by activating innate antitumor immunity, which includes the stimulator of interferon genes (STING) pathway. Research on mice has investigated the intricate relationship between STING signaling and the tumor immune microenvironment (TIME), revealing new modes of action such as NK cells and contrasting the significance of STING activation in tumor cells with immune cells [108,109,110]. Treating tissue fragment explants and patient-derived organotypic tumor spheroids (PDOTS) in short-term culture facilitates the development of immunological treatments as a result of advancements in the study of the human TIME using patient samples [111,112].

These platforms can track patient response and have been primarily utilized to study anti-PD(L)-1 immune checkpoint drugs, but they also hold potential for the development of new cancer immunotherapies. Recent research has revealed that a number of human cancer types suppress STING and the downstream interferon response in order to evade immune identification, highlighting the critical role tumor-cell STING signaling plays in human cancer [113,114]. However, the effects of STING agonists on tumor cells and other cell types in the human TIME have not been thoroughly studied. This knowledge could guide the creation of innovative treatment combinations, such as cell therapy.

It might be incredibly helpful to perform a large-scale study utilizing PDOTS and establishing a methodology to analyze the STING agonist response in an inflammatory histotype and perform dynamic single-cell RNA sequencing in tumor explants [115]. Erik H. Knelson et al.’s 2022 study found that increased tumor-cell STING expression in MPM makes patients more susceptible to therapeutic STING agonism, particularly in combination with NK-cell therapy [115]. The quick clearance of injectable drugs and their short therapeutic window constrain the clinical development of STING agonists [116].

The current data available suggest that continuous exposure is likely to kill endogenous effector T cells and restrict combinations with adoptively transferred transgenic TCR T or CAR T-cell therapies, even though novel slow-release and systemic formulations of STING agonists could address some of these problems [117]. Knelson’s study revealed that NK cells are activated and recruited to kill MPM cells in spite of their resistance to continuous high-dose STING agonist exposure. This finding supports the novel immunobiology and offers a combinatorial strategy for the development of NK-cell therapies in clinical settings [115].

As an alternative, adoptive T-cell treatments could be modified to thwart the cytotoxicity of STING agonists by reestablishing efficient autophagy or blocking the STING pathway. Studying innate/adaptive immune crosstalk and how activating one channel affects the larger TIME using tumor samples from patients may help determine the best ways to advance developing cell treatments and prevent immune depletion.

## 6. Novel Insights

### 6.1. Targeting PM Proteomic Profile

It is critical to determine the molecular processes that initiate and drive the growth of this tumor to create novel therapeutic approaches. A meta-analysis on the genes and pathways connected to the development of pleural mesothelioma was released in June 2023. To obtain insight into the molecular mechanisms underpinning these genes, differentially expressed genes (DEGs) and protein–protein interaction networks (PPINs) were found via functional enrichment analysis.

Additionally, the authors forecasted the minimal medication inhibitory concentration of an anticancer agent for MPM and built survival prediction models for certain DEGs. A total of 26 pleural tissue controls and 115 PM tumor transcriptomes were examined. In total, 1046 DEGs that were increased were found in the PM samples. Tumor sample cellular signaling categories were linked to the AMPK, PI3KAkt, and TNF pathways. Biological processes that were overrepresented included the regulation of angiogenesis, the migration of cells, and the inflammatory response. Reduced survival rates were linked to SOX17 and TACC1 expression.

The development of this kind of tumor is closely correlated with the cellular reaction to chemical carcinogens, primarily asbestos. Alterations in microsomal glutathione transferase 2 (MGST2) may result in the generation of reactive oxygen species (ROS) and leukotriene C4, a mediator of inflammation [118]. This study demonstrated that the MGST2 gene was elevated in MPM tumors. Even though the majority of the pathways found in this meta-analysis—like MAPK, NK-kB, pi3k, Akt, and TNF—are also relevant to other types of cancer, the analysis opens up new research directions for PM.

For instance, research on LPL’s role in sustaining the tumor’s supply of fatty nutrients has been carried out on lung cancer, but not on PM [119]. Remarkably, this aligns with more recent data indicating a lipid metabolic reprogramming in the course of mesothelioma [120]. Interestingly, one obvious characteristic of aggressive tumors is the amplification of lipid droplets. Lipophilic anticancer treatments include targeting lipoid microenvironment compartments or the lipid metabolism [121].

The authors also highlighted licensed medications used in other neoplasms and disorders that could be suggested as treatment options for MPM based on the top elevated DEGs from the meta-analysis. The expression of SOX17, one of the most highly expressed genes in MPM tumors, was linked to a lower survival rate in MPM patients. The scientists looked at the connection between SOX17 expression and the tumors’ susceptibility to anticancer medications. Significantly, their research revealed that in MPM patients, the SOX17 expression level may influence medication susceptibility.

Particularly, CMK, Docetaxel, and Rapamycin demonstrated differential IC50 sensitivity in the SOX17 high-expression group compared to Erlotinib, Lapatinib, and Mitomycin C in the SOX17 low-expression group. These results imply that in PM patients, the SOX17 expression level may function as a predictive biomarker for medication sensitivity. In tumor development and repression, SOX17 has conflicting roles. On the one hand, SOX17 is a transcription factor that is essential for the development of endoderm during embryogenesis.

Dysregulation of SOX17 is a major component in the development and progression of numerous types of cancer, including endometrial, esophageal, and breast. A low expression of SOX17 in certain cancer types has been linked to a bad prognosis for the patient. However, by upregulating VEGFR2 and improving the recruitment of myeloid-derived suppressor cells, which promotes vascular remodeling and reduces the production of IFN-γ and Th2 cytokines, SOX17 may potentially boost tumor angiogenesis and blood vessel instability [122].

Furthermore, endometrial cancers with hyperbranched and hyperdense vascular tumors expressed more SOX17; SOX17 deletion suppressed angiogenesis in tumors and restored normalcy to tumor vasculature. Conversely, patients with primary gastric cancer have a worse chance of surviving when their TACC1 expression is elevated. Through microtubule stability in the developing spindle, the TACC1 protein is involved in the regulation of mitotic checkpoints. TACC1 has the ability to transform fibroblasts and, as evidenced by current research, positively regulates the Ras and PI3K signaling pathways to promote transformation and mammary carcinogenesis. The progression of non-small cell lung cancer is halted by the microRNA miR-4742-5p, which inhibits TACC1 expression [123].

### 6.2. Exploiting PM-OMIC Profile

The resolution of PM heterogeneity was significantly increased by omics profiling, indicating that this disease cannot be precisely classified into distinct entities but should be viewed as a continuum between the two extreme morphological forms. For the first time, a morphology guided spatial transcriptomic approach was applied to a particular series of B-MPMs in a work published in 2023 by Torricelli et al. This approach allowed for the collection of gene expression data in a spatial context, which, in turn, allowed for the provision of additional details regarding the process of MPM progression [124]. The information gathered painted a picture of a complex ecosystem in which immune cells and MPM interact to change the local microenvironment and promote development.

From the very beginning of cellular transdifferentiation, the S morphology is characterized by a robust inflammatory milieu. In the transitional zones, it was found that the S components were linked to elevated inflammatory cytokine expression and significant inflammatory cell recruitment. These signals encourage the activation of both innate and adaptive immunity, which, in turn, directs many effector cells—particularly CD8+T-cells—to target the tumor locations. Yet, as indicated by the elevated expression levels of immunological checkpoints such as the T-cell receptor HAVCR2 and its particular ligand LGASL9, these S-associated immune cells are driven to fatigue by the ongoing activation by tumor antigens and the endurance of the inflammatory stimuli.

PM cells experience a significant structural rearrangement that is driven by transcription. E-PM cells lose their epithelial characteristics, profoundly altering their capacity to interact with their surroundings, driven by the production of TFs linked to EMT. Remodeling factors and proteins that interact with the matrix take the place of cell-to-cell adhesion structures. ECM is an essential component of the ecosystem around cancer, and its dynamic alterations play a significant role in the etiology and development of the illness [125]. ECM’s stiffness and adhesive cues provide mechanical signals to cancer cells, which are mainly transduced by cytoskeleton-anchored proteins. These signals influence gene expression by supporting programs that help cells adapt to external stimuli by changing cell architecture, fostering motility, and overcoming mechanically induced stresses [126].

Aberrant oxidative stress is one of the several stress stimuli that cause ECM remodeling. The inhalation of asbestos fibers results in the formation of free radicals and the ongoing release of reactive species. These extremely dynamic components have the potential to induce significant molecular instability and work together to change ECM as MPM advances [127]. According to recent findings, the extracellular matrix (ECM) plays a significant immune-modulatory role in cancer by forming niches that regulate the migration, localization, phenotype, and function of immune cells that infiltrate tumors. This, in turn, helps the immune system evade detection and treatment [128].

Furthermore, a recent study on pancreatic cancer has demonstrated that the transcriptional program associated with the extracellular matrix (ECM) is correlated with TGFB signaling and may be connected with immune evasion or adaptability [129]. Torricelli et al. used gene expression data from their internal cohort of MPMs to show that TGFB1 is a sign of clinical aggressiveness in PM and is linked to a worse chance of survival. This may have significant effects on how patients are managed. The authors also discovered that the main targets of TGFB1 in PM are TAMs, or tumor-associated macrophages. In addition to causing inflammation and tissue remodeling, M2-TAMs have been shown to induce T-cell inhibitory immunological checkpoints by establishing an immunosuppressive milieu [130].

These data show that M2-TAMs are predominant in the immunological milieu of B-MPM and that there is a strong association between M2-TAM markers and immune checkpoints that are specific to T cells. The role of M2-TAM in PMs is being brought to light by a number of recent studies that employ radically different methodologies. For example, Ollila et al. used multiplexed fluorescence immunohistochemistry to find that M2-TAMs are independently linked to a shorter survival in a retrospective cohort of E-PMs [131]. Using a thorough genomic immune profiling, Creaney et al. demonstrated that the PM immunological milieu had elevated amounts of M2-TAMs linked to the expression of TGFB1 and metalloproteinases (MMP2 and MMP14) [132].

Macrophages from PM patients’ pleural effusions inhibit the antitumor T cell immunological response, as demonstrated by Lievense et al. [133]. On the other hand, using a CSF-1R kinase inhibitor to pharmacologically deplete M2-TAM improves the efficiency of dendritic cell vaccine treatment for priming anticancer immunity [134]. These findings highlight this population’s contribution to PM and draw attention to the possibility of targeting macrophages. Naturally, there are a few significant restrictions; first and foremost, this is a descriptive transcriptome study that only looks at gene transcripts. This is the first paper to investigate B-PM using a spatial transcriptome approach.

Within this framework, the work described provides fresh, pertinent viewpoints: it establishes for the first time the functional significance of the immune system and the inflammatory milieu in the mechanisms driving PM evolution; it also uncovers new information regarding the topography of the lesion and the communication circuits between immune cells and tumors, thereby offering new candidates to test as predictive biomarkers of ICI response. Furthermore, it suggests that M2-TAM polarization plays a crucial role in the development of immune evasion signals, identifying fresh possible targets [135].

### 6.3. Genomic Basis of Novel Trials

Since chromosomal losses and changes are common, while activating mutations are uncommon, in PM, the question of alternate treatments to the present standards remains unresolved. Specifically, a significant proportion (5–10%) of PM patients with germline mutations in the DDR genes pathway have demonstrated a pertinent sensitivity to asbestos, while *BRCA1/2* and other genes implicated in the HRR route have been found to be mutated in a large number of PM patients [136]. Furthermore, somatic inactivating mutations in the BRCA-1-related gene (BAP1) were found in over 20% of PM, and these mutations appear to be crucial in the pathophysiology of tumors [137].

These theories have given rise to the notion that cells expressing mutant *BAP1* would need the PARP-1 enzyme to survive, which has prompted the start of multiple preclinical investigations examining the possible effectiveness of PARPi in this situation [138]. Interestingly, two distinct preclinical studies have demonstrated that both niraparib and olaparib have potential efficacy against PM cells, regardless of the presence of BAP1 mutations, largely eliminating the possible link between the BAP1 gene and PARP enzyme specific inhibition [139,140]. The effectiveness and tolerability of PARPi in MPM have been assessed in numerous trials; nevertheless, the PFS and OS results were largely negative.

One of the ongoing clinical trials is UNITO-001, a prospective phase II single-arm study that assesses the efficacy of dostarlimab and niraparib in metastatic mesotheliomas that have both positive PD-L1 (TPS > 1 percent) and HRR insufficiency [141].

PFS, toxicity, and safety evaluation are the secondary goals. The key endpoint is the non-progression proportion, which is the percentage of patients free of progression six months after talazoparib initiation. The MiST trial is a phase II, single-arm research designed to evaluate rucaparib’s effectiveness in patients with PM who have a *BAP1* or *BRCA* mutation. DCR is the main goal, whereas ORR, toxicity, and safety evaluation are the secondary endpoints [142]. Furthermore, mesothelioma with homologous recombination defect linked to a *BRCA1* mutation has given rise to new PARPi research. In many solid tumors, PARPi are a concrete fact. Several trials are investigating their possible use in conditions including thoracic malignancies, where they may be a unique treatment alternative.

These medications do, however, still have several significant drawbacks, such as resistance mechanisms and unanticipated side effects. Additionally, more research is needed to completely establish these medications’ efficacy as single agents in the setting of thoracic malignancies. Given the data at hand, there is increasing interest in investigating the possible synergistic effect and, as a result, combining the administration of PARPi and PARGi with other treatments such as radiotherapy, chemotherapy, or immunotherapy. New approaches, like NGS tumor profiling, may prove useful in identifying trustworthy predictors that direct patient selection in clinical settings. Furthermore, off-site toxicity or drug resistance may be lessened in the next years due to the recent development of nanomedicine, creating new and intriguing opportunities for PARPi and PARGi in clinical practice [143].

### 6.4. Interfering with Cell Invasive Properties

It has been demonstrated in other current and encouraging research that YB-1, or Y-box-binding protein 1, stimulates cell migration and proliferation in PM. Schlech (2023) assessed the impact of YB-1’s genetic and pharmacological targeting on PM proliferation and responsiveness to radiation and cisplatin treatment. Reduced cell proliferation following YB-1 knockdown via siRNA was found to be substantially linked with wt BAP1 and mutant NF2 and P53 status. In 20 PM cell lines, YB-1 knockdown-induced growth inhibition was associated with the effectiveness of entinostat in inhibiting YB-1 deacetylation. Both entinostat and siRNA have been shown to decrease tumor growth in animal xenotransplant models. Moreover, entinostat and YBX1-targeting siRNA increased radiation and cisplatin sensitivity.

Specifically, in all examined cell models, entinostat demonstrated robust synergistic interactions with cisplatin, which were associated with a markedly enhanced cellular absorption of platinum. Crucially, cisplatin with entinostat together produced a greater growth suppression in a mouse model than either medication alone. In PM, YB-1 may be a desirable target, and using an entinostat to target YB-1 is a viable strategy to increase radiation and cisplatin sensitivity [138]. However, the direct targeting of YB-1 in clinical contexts is difficult since it lacks a kinase domain and other enzymatic activities.

Preclinical research has shown that RNA-mediated silencing of YB-1 is simple, and clinical studies have shown that RNA-based therapies can be delivered to treat a variety of cancers, including PM [144]. In trials for non-small-cell lung cancer, entinostat has also been used in conjunction with a number of other medications, such as the EGFR inhibitor erlotinib and the DNA methylation inhibitor azacitidine [145,146]. It was discovered that entinostat, a class I HDAC inhibitor, increased histone acetylation and the expression of p21 and PTEN, two tumor suppressor genes [147]. More recently, it was found that entinostat might raise YB-1 acetylation in sarcoma cells, where more YB-1 acetylation, particularly of lysine 81 (K81), significantly prevented metastasis [148].

Schlech verified that entinostat IC50 values coincide with YB-1 siRNA’s growth inhibitory activity, indicating that YB-1 targeting significantly contributes to entinostat’s overall effectiveness in PM. However, YB-1-independent mechanisms, such the previously reported upregulation of PTEN and p21 expression, may also play a role in the antineoplastic impact against PM cells [138]. This article reported for the first time that YB-1 hyperacetylation may play a role in entinostat’s efficacy in cancer types other than sarcoma and PM; future studies employing this chemical should take this possibility into account.

According to recent evidence, entinostat may improve the effects of immune checkpoint inhibitors (ICIs) in models of bladder and breast cancer by influencing the tumor microenvironment or immune-editing tumor neoantigens [149,150]. Although it has not been proven, YB-1 may have a role in entinostat’s ability to enhance ICI effects. This could lead to intriguing new treatments for PM patients. YB-1 knockdown and entinostat treatment demonstrated additive to synergistic benefits when paired with cisplatin or radiation therapy. Overall, Schelch’s research indicates that YB-1 may be a worthwhile target for MPM. Furthermore, it was demonstrated that using entinostat to target YB-1 pharmacologically is viable and has the potential to interact synergistically with radiation or cisplatin, which justifies future investigation of the drug as a component of therapy regimens [138].

## 7. Conclusions

Biomolecular heterogeneity is a key feature of PM, which does not display key genetic drivers. This characteristic has impaired the development of personalized approaches against PM. However, the development of high-throughput technologies and OMIC platforms has allowed for a rapidly increasing number of biologic and genomic data exploitable in the clinical arena. The integration of more fields of expertise has proven to be fundamental in every step concerning this disease, providing a prompt and comprehensive evaluation of at-risk subjects, and the most appropriate choice of treatment. It is thus clearly suggested that PM patients should be referred to specialized centers and be evaluated by interdisciplinary teams to assess the most optimal strategy tailored to the patient.

## References

[1] Gaudino G., Xue J., Yang H. (2020). How Asbestos and Other Fibers Cause Mesothelioma. Transl. Lung Cancer Res..

[2] Abbott D.M., Bortolotto C., Benvenuti S., Lancia A., Filippi A.R., Stella G.M. (2020). Malignant Pleural Mesothelioma: Genetic and Microenviromental Heterogeneity as an Unexpected Reading Frame and Therapeutic Challenge. Cancers.

[3] Driscoll T., Nelson D.I., Steenland K., Leigh J., Concha-Barrientos M., Fingerhut M., Prüss-Ustün A. (2005). The Global Burden of Disease Due to Occupational Carcinogens. Am. J. Ind. Med..

[4] Delgermaa V., Takahashi K., Park E.K., Le G.V., Hara T., Sorahan T. (2011). Les Décés Mondiaux Par Mésothéliome Rapportés á l’Organisation Mondiale de La Santé Entre 1994 et 2008. Bull. World Health Organ..

[5] Popat S., Baas P., Faivre-Finn C., Girard N., Nicholson A.G., Nowak A.K., Opitz I., Scherpereel A., Reck M. (2022). Malignant Pleural Mesothelioma: ESMO Clinical Practice Guidelines for Diagnosis, Treatment and Follow-Up☆. Ann. Oncol..

[6] Alpert N., van Gerwen M., Taioli E. (2020). Epidemiology of Mesothelioma in the 21st Century in Europe and the United States, 40 Years after Restricted/Banned Asbestos Use. Transl. Lung Cancer Res..

[7] Jiang Z., Chen T., Chen J., Ying S., Gao Z., He X., Miao C., Yu M., Feng L., Xia H. (2018). Hand-Spinning Chrysotile Exposure and Risk of Malignant Mesothelioma: A Case–Control Study in Southeastern China. Int. J. Cancer.

[8] Schumann S.O., Kocher G., Minervini F. (2021). Epidemiology, Diagnosis and Treatment of the Malignant Pleural Mesothelioma, a Narrative Review of Literature. J. Thorac. Dis..

[9] Cohen-Mansfield J., Dakheel-Ali M., Marx M.S., Thein K., Regier N.G., Waage P. (2017). HHS Public Access. Physiol. Behav..

[10] Baumann F., Ambrosi J.-P., Carbone M. (2013). Asbestos Is Not Just Asbestos: An Unrecognised Health Hazard. Lancet. Oncol..

[11] Carbone M., Emri S., Dogan A.U., Steele I., Tuncer M., Pass H.I., Baris Y.I. (2007). A Mesothelioma Epidemic in Cappadocia: Scientific Developments and Unexpected Social Outcomes. Nat. Rev. Cancer.

[12] Stanton M.F., Laynard M., Tegeris A., Miller E., May M., Kent E. (1977). Carcinogenicity of Fibrous Glass: Pleural Response in the Rat in Relation to Fiber Dimension. J. Natl. Cancer Inst..

[13] Huang S.X.L., Jaurand M.-C., Kamp D.W., Whysner J., Hei T.K. (2011). Role of Mutagenicity in Asbestos Fiber-Induced Carcinogenicity and Other Diseases. J. Toxicol. Environ. Health. B Crit. Rev..

[14] Mossman B.T. (1990). In Vitro Studies on the Biologic Effects of Fibers: Correlation with in Vivo Bioassays. Environ. Health Perspect..

[15] Barlow C.A., Grespin M., Best E.A. (2017). Asbestos Fiber Length and Its Relation to Disease Risk. Inhal. Toxicol..

[16] Carbone M., Yang H. (2012). Molecular Pathways: Targeting Mechanisms of Asbestos and Erionite Carcinogenesis in Mesothelioma. Clin. Cancer Res. Off. J. Am. Assoc. Cancer Res..

[17] Bernstein D.M., Donaldson K., Decker U., Gaering S., Kunzendorf P., Chevalier J., Holm S.E. (2008). A Biopersistence Study Following Exposure to Chrysotile Asbestos Alone or in Combination with Fine Particles. Inhal. Toxicol..

[18] Qi F., Okimoto G., Jube S., Napolitano A., Pass H.I., Laczko R., Demay R.M., Khan G., Tiirikainen M., Rinaudo C. (2013). Continuous Exposure to Chrysotile Asbestos Can Cause Transformation of Human Mesothelial Cells via HMGB1 and TNF-α Signaling. Am. J. Pathol..

[19] Larson D., Powers A., Ambrosi J.-P., Tanji M., Napolitano A., Flores E.G., Baumann F., Pellegrini L., Jennings C.J., Buck B.J. (2016). Investigating Palygorskite’s Role in the Development of Mesothelioma in Southern Nevada: Insights into Fiber-Induced Carcinogenicity. J. Toxicol. Environ. Health. B Crit. Rev..

[20] Carbone M., Adusumilli P.S., Alexander H.R.J., Baas P., Bardelli F., Bononi A., Bueno R., Felley-Bosco E., Galateau-Salle F., Jablons D. (2019). Mesothelioma: Scientific Clues for Prevention, Diagnosis, and Therapy. CA. Cancer J. Clin..

[21] Nagai H., Ishihara T., Lee W.-H., Ohara H., Okazaki Y., Okawa K., Toyokuni S. (2011). Asbestos Surface Provides a Niche for Oxidative Modification. Cancer Sci..

[22] Xu A., Wu L.J., Santella R.M., Hei T.K. (1999). Role of Oxyradicals in Mutagenicity and DNA Damage Induced by Crocidolite Asbestos in Mammalian Cells. Cancer Res..

[23] Toyokuni S. (2019). Iron Addiction with Ferroptosis-Resistance in Asbestos-Induced Mesothelial Carcinogenesis: Toward the Era of Mesothelioma Prevention. Free Radic. Biol. Med..

[24] Ramos-Nino M.E., Blumen S.R., Sabo-Attwood T., Pass H., Carbone M., Testa J.R., Altomare D.A., Mossman B.T. (2008). HGF Mediates Cell Proliferation of Human Mesothelioma Cells through a PI3K/MEK5/Fra-1 Pathway. Am. J. Respir. Cell Mol. Biol..

[25] Broaddus V.C., Yang L., Scavo L.M., Ernst J.D., Boylan A.M. (1996). Asbestos Induces Apoptosis of Human and Rabbit Pleural Mesothelial Cells via Reactive Oxygen Species. J. Clin. Investig..

[26] Yang H., Bocchetta M., Kroczynska B., Elmishad A.G., Chen Y., Liu Z., Bubici C., Mossman B.T., Pass H.I., Testa J.R. (2006). TNF-Alpha Inhibits Asbestos-Induced Cytotoxicity via a NF-KappaB-Dependent Pathway, a Possible Mechanism for Asbestos-Induced Oncogenesis. Proc. Natl. Acad. Sci. USA.

[27] Carbone M., Yang H. (2017). Mesothelioma: Recent Highlights. Ann. Transl. Med..

[28] Yang H., Rivera Z., Jube S., Nasu M., Bertino P., Goparaju C., Franzoso G., Lotze M.T., Krausz T., Pass H.I. (2010). Programmed Necrosis Induced by Asbestos in Human Mesothelial Cells Causes High-Mobility Group Box 1 Protein Release and Resultant Inflammation. Proc. Natl. Acad. Sci. USA.

[29] Kadariya Y., Menges C.W., Talarchek J., Cai K.Q., Klein-Szanto A.J., Pietrofesa R.A., Christofidou-Solomidou M., Cheung M., Mossman B.T., Shukla A. (2016). Inflammation-Related IL1β/IL1R Signaling Promotes the Development of Asbestos-Induced Malignant Mesothelioma. Cancer Prev. Res..

[30] Thompson J.K., Shukla A., Leggett A.L., Munson P.B., Miller J.M., MacPherson M.B., Beuschel S.L., Pass H.I., Shukla A. (2018). Extracellular Signal Regulated Kinase 5 and Inflammasome in Progression of Mesothelioma. Oncotarget.

[31] Pellegrini L., Xue J., Larson D., Pastorino S., Jube S., Forest K.H., Saad-Jube Z.S., Napolitano A., Pagano I., Negi V.S. (2017). HMGB1 Targeting by Ethyl Pyruvate Suppresses Malignant Phenotype of Human Mesothelioma. Oncotarget.

[32] Jube S., Rivera Z.S., Bianchi M.E., Powers A., Wang E., Pagano I., Pass H.I., Gaudino G., Carbone M., Yang H. (2012). Cancer Cell Secretion of the DAMP Protein HMGB1 Supports Progression in Malignant Mesothelioma. Cancer Res..

[33] Affar E.B., Carbone M. (2018). BAP1 Regulates Different Mechanisms of Cell Death. Cell Death Dis..

[34] Carbone M., Amelio I., Affar E.B., Brugarolas J., Cannon-Albright L.A., Cantley L.C., Cavenee W.K., Chen Z., Croce C.M., Andrea A.D. (2018). Consensus Report of the 8 and 9th Weinman Symposia on Gene x Environment Interaction in Carcinogenesis: Novel Opportunities for Precision Medicine. Cell Death Differ..

[35] Ly P., Cleveland D.W. (2017). Rebuilding Chromosomes After Catastrophe: Emerging Mechanisms of Chromothripsis. Trends Cell Biol..

[36] Yoshikawa Y., Emi M., Hashimoto-Tamaoki T., Ohmuraya M., Sato A., Tsujimura T., Hasegawa S., Nakano T., Nasu M., Pastorino S. (2016). High-Density Array-CGH with Targeted NGS Unmask Multiple Noncontiguous Minute Deletions on Chromosome 3p21 in Mesothelioma. Proc. Natl. Acad. Sci. USA.

[37] Oey H., Daniels M., Relan V., Chee T.M., Davidson M.R., Yang I.A., Ellis J.J., Fong K.M., Krause L., Bowman R. (2019). V Whole-Genome Sequencing of Human Malignant Mesothelioma Tumours and Cell Lines. Carcinogenesis.

[38] Mansfield A.S., Peikert T., Smadbeck J.B., Udell J.B.M., Garcia-Rivera E., Elsbernd L., Erskine C.L., Van Keulen V.P., Kosari F., Murphy S.J. (2019). Neoantigenic Potential of Complex Chromosomal Rearrangements in Mesothelioma. J. Thorac. Oncol. Off. Publ. Int. Assoc. Study Lung Cancer.

[39] Husain A.N., Colby T.V., Ordóñez N.G., Allen T.C., Attanoos R.L., Beasley M.B., Butnor K.J., Chirieac L.R., Churg A.M., Dacic S. (2018). Guidelines for Pathologic Diagnosis of Malignant Mesothelioma 2017 Update of the Consensus Statement From the International Mesothelioma Interest Group. Arch. Pathol. Lab. Med..

[40] Jongsma J., van Montfort E., Vooijs M., Zevenhoven J., Krimpenfort P., van der Valk M., van de Vijver M., Berns A. (2008). A Conditional Mouse Model for Malignant Mesothelioma. Cancer Cell.

[41] Altomare D.A., Menges C.W., Pei J., Zhang L., Skele-Stump K.L., Carbone M., Kane A.B., Testa J.R. (2009). Activated TNF-Alpha/NF-KappaB Signaling via down-Regulation of Fas-Associated Factor 1 in Asbestos-Induced Mesotheliomas from Arf Knockout Mice. Proc. Natl. Acad. Sci. USA.

[42] Sato T., Sekido Y. (2018). NF2/Merlin Inactivation and Potential Therapeutic Targets in Mesothelioma. Int. J. Mol. Sci..

[43] Altomare D.A., Vaslet C.A., Skele K.L., De Rienzo A., Devarajan K., Jhanwar S.C., McClatchey A.I., Kane A.B., Testa J.R. (2005). A Mouse Model Recapitulating Molecular Features of Human Mesothelioma. Cancer Res..

[44] Rehrauer H., Wu L., Blum W., Pecze L., Henzi T., Serre-Beinier V., Aquino C., Vrugt B., de Perrot M., Schwaller B. (2018). How Asbestos Drives the Tissue towards Tumors: YAP Activation, Macrophage and Mesothelial Precursor Recruitment, RNA Editing, and Somatic Mutations. Oncogene.

[45] Wiesner T., Obenauf A.C., Murali R., Fried I., Griewank K.G., Ulz P., Windpassinger C., Wackernagel W., Loy S., Wolf I. (2011). Germline Mutations in BAP1 Predispose to Melanocytic Tumors. Nat. Genet..

[46] Bononi A., Giorgi C., Patergnani S., Larson D., Verbruggen K., Tanji M., Pellegrini L., Signorato V., Olivetto F., Pastorino S. (2017). BAP1 Regulates IP3R3-Mediated Ca(2+) Flux to Mitochondria Suppressing Cell Transformation. Nature.

[47] Testa J.R., Cheung M., Pei J., Below J.E., Tan Y., Sementino E., Cox N.J., Dogan A.U., Pass H.I., Trusa S. (2011). Germline BAP1 Mutations Predispose to Malignant Mesothelioma. Nat. Genet..

[48] Bononi A., Yang H., Giorgi C., Patergnani S., Pellegrini L., Su M., Xie G., Signorato V., Pastorino S., Morris P. (2017). Germline BAP1 Mutations Induce a Warburg Effect. Cell Death Differ..

[49] Finn R.S., Brims F.J.H., Gandhi A., Olsen N., Musk A.W., Maskell N.A., Lee Y.C.G. (2012). Postmortem Findings of Malignant Pleural Mesothelioma: A Two-Center Study of 318 Patients. Chest.

[50] Cardinale L., Ardissone F., Gned D., Sverzellati N., Piacibello E., Veltri A. (2017). Diagnostic Imaging and Workup of Malignant Pleural Mesothelioma. Acta Biomed..

[51] Bianco A., Valente T., de Rimini M.L., Sica G., Fiorelli A. (2018). Clinical Diagnosis of Malignant Pleural Mesothelioma. J. Thorac. Dis..

[52] Patz E.F.J., Shaffer K., Piwnica-Worms D.R., Jochelson M., Sarin M., Sugarbaker D.J., Pugatch R.D. (1992). Malignant Pleural Mesothelioma: Value of CT and MR Imaging in Predicting Resectability. AJR Am. J. Roentgenol..

[53] Heelan R.T., Rusch V.W., Begg C.B., Panicek D.M., Caravelli J.F., Eisen C. (1999). Staging of Malignant Pleural Mesothelioma: Comparison of CT and MR Imaging. AJR Am. J. Roentgenol..

[54] Sandach P., Seifert R., Fendler W.P., Hautzel H., Herrmann K., Maier S., Plönes T., Metzenmacher M., Ferdinandus J. (2022). A Role for PET/CT in Response Assessment of Malignant Pleural Mesothelioma. Semin. Nucl. Med..

[55] Yildirim H., Metintas M., Entok E., Ak G., Ak I., Dundar E., Erginel S. (2009). Clinical Value of Fluorodeoxyglucose-Positron Emission Tomography/Computed Tomography in Differentiation of Malignant Mesothelioma from Asbestos-Related Benign Pleural Disease: An Observational Pilot Study. J. Thorac. Oncol. Off. Publ. Int. Assoc. Study Lung Cancer.

[56] Frauenfelder T., Kestenholz P., Hunziker R., Nguyen T.D.L., Fries M., Veit-Haibach P., Husmann L., Stahel R., Weder W., Opitz I. (2015). Use of Computed Tomography and Positron Emission Tomography/Computed Tomography for Staging of Local Extent in Patients with Malignant Pleural Mesothelioma. J. Comput. Assist. Tomogr..

[57] Basu S., Saboury B., Torigian D.A., Alavi A. (2011). Current Evidence Base of FDG-PET/CT Imaging in the Clinical Management of Malignant Pleural Mesothelioma: Emerging Significance of Image Segmentation and Global Disease Assessment. Mol. Imaging Biol..

[58] Sharif S., Zahid I., Routledge T., Scarci M. (2011). Does Positron Emission Tomography Offer Prognostic Information in Malignant Pleural Mesothelioma?. Interact. Cardiovasc. Thorac. Surg..

[59] Yamamuro M., Gerbaudo V.H., Gill R.R., Jacobson F.L., Sugarbaker D.J., Hatabu H. (2007). Morphologic and Functional Imaging of Malignant Pleural Mesothelioma. Eur. J. Radiol..

[60] Qureshi N.R., Rahman N.M., Gleeson F.V. (2009). Thoracic Ultrasound in the Diagnosis of Malignant Pleural Effusion. Thorax.

[61] Henderson D.W., Reid G., Kao S.C., van Zandwijk N., Klebe S. (2013). Challenges and Controversies in the Diagnosis of Mesothelioma: Part 1. Cytology-Only Diagnosis, Biopsies, Immunohistochemistry, Discrimination between Mesothelioma and Reactive Mesothelial Hyperplasia, and Biomarkers. J. Clin. Pathol..

[62] Baas P., Fennell D., Kerr K.M., Van Schil P.E., Haas R.L., Peters S. (2015). Malignant Pleural Mesothelioma: ESMO Clinical Practice Guidelines for Diagnosis, Treatment and Follow-Up. Ann. Oncol. Off. J. Eur. Soc. Med. Oncol..

[63] Nowak A.K., Chansky K., Rice D.C., Pass H.I., Kindler H.L., Shemanski L., Billé A., Rintoul R.C., Batirel H.F., Thomas C.F. (2016). The IASLC Mesothelioma Staging Project: Proposals for Revisions of the T Descriptors in the Forthcoming Eighth Edition of the TNM Classification for Pleural Mesothelioma. J. Thorac. Oncol. Off. Publ. Int. Assoc. Study Lung Cancer.

[64] Rice D., Chansky K., Nowak A., Pass H., Kindler H., Shemanski L., Opitz I., Call S., Hasegawa S., Kernstine K. (2016). The IASLC Mesothelioma Staging Project: Proposals for Revisions of the N Descriptors in the Forthcoming Eighth Edition of the TNM Classification for Pleural Mesothelioma. J. Thorac. Oncol. Off. Publ. Int. Assoc. Study Lung Cancer.

[65] Bibby A.C., Tsim S., Kanellakis N., Ball H., Talbot D.C., Blyth K.G., Maskell N.A., Psallidas I. (2016). Malignant Pleural Mesothelioma: An Update on Investigation, Diagnosis and Treatment. Eur. Respir. Rev. Off. J. Eur. Respir. Soc..

[66] Sauter J.L., Dacic S., Galateau-Salle F., Attanoos R.L., Butnor K.J., Churg A., Husain A.N., Kadota K., Khoor A., Nicholson A.G. (2022). The 2021 WHO Classification of Tumors of the Pleura: Advances Since the 2015 Classification. J. Thorac. Oncol. Off. Publ. Int. Assoc. Study Lung Cancer.

[67] Mastromarino M.G., Lenzini A., Aprile V., Alì G., Bacchin D., Korasidis S., Ambrogi M.C., Lucchi M. (2022). New Insights in Pleural Mesothelioma Classification Update: Diagnostic Traps and Prognostic Implications. Diagnostics.

[68] Hysi I., Le Pimpec-Barthes F., Alifano M., Venissac N., Mouroux J., Regnard J.-F., Riquet M., Porte H. (2014). Lymph Node Involvement and Metastatic Lymph Node Ratio Influence the Survival of Malignant Pleural Mesothelioma: A French Multicenter Retrospective Study. Oncol. Rep..

[69] Berzenji L., Van Schil P.E., Carp L. (2018). The Eighth TNM Classification for Malignant Pleural Mesothelioma. Transl. Lung Cancer Res..

[70] Lim E., Darlison L., Edwards J., Elliott D., Fennell D.A., Popat S., Rintoul R.C., Waller D., Ali C., Bille A. (2020). Mesothelioma and Radical Surgery 2 (MARS 2): Protocol for a Multicentre Randomised Trial Comparing (Extended) Pleurectomy Decortication versus No (Extended) Pleurectomy Decortication for Patients with Malignant Pleural Mesothelioma. BMJ Open.

[71] Treasure T., Lang-Lazdunski L., Waller D., Bliss J.M., Tan C., Entwisle J., Snee M., O’Brien M., Thomas G., Senan S. (2011). Extra-Pleural Pneumonectomy versus No Extra-Pleural Pneumonectomy for Patients with Malignant Pleural Mesothelioma: Clinical Outcomes of the Mesothelioma and Radical Surgery (MARS) Randomised Feasibility Study. Lancet Oncol..

[72] Rice D., Rusch V., Pass H., Asamura H., Nakano T., Edwards J., Giroux D.J., Hasegawa S., Kernstine K.H., Waller D. (2011). Recommendations for Uniform Definitions of Surgical Techniques for Malignant Pleural Mesothelioma: A Consensus Report of the International Association for the Study of Lung Cancer International Staging Committee and the International Mesothelioma Interes. J. Thorac. Oncol. Off. Publ. Int. Assoc. Study Lung Cancer.

[73] Flores R.M., Pass H.I., Seshan V.E., Dycoco J., Zakowski M., Carbone M., Bains M.S., Rusch V.W. (2008). Extrapleural Pneumonectomy versus Pleurectomy/Decortication in the Surgical Management of Malignant Pleural Mesothelioma: Results in 663 Patients. J. Thorac. Cardiovasc. Surg..

[74] Rusch V.W., Giroux D., Kennedy C., Ruffini E., Cangir A.K., Rice D., Pass H., Asamura H., Waller D., Edwards J. (2012). Initial Analysis of the International Association for the Study of Lung Cancer Mesothelioma Database. J. Thorac. Oncol. Off. Publ. Int. Assoc. Study Lung Cancer.

[75] Sugarbaker D.J., Richards W.G., Bueno R. (2014). Extrapleural Pneumonectomy in the Treatment of Epithelioid Malignant Pleural Mesothelioma: Novel Prognostic Implications of Combined N1 and N2 Nodal Involvement Based on Experience in 529 Patients. Ann. Surg..

[76] Kindler H.L., Ismaila N., Armato S.G., Bueno R., Hesdorffer M., Jahan T., Jones C.M., Miettinen M., Pass H., Rimner A. (2018). Treatment of Malignant Pleural Mesothelioma: American Society of Clinical Oncology Clinical Practice Guideline. J. Clin. Oncol. Off. J. Am. Soc. Clin. Oncol..

[77] McMillan R.R., Berger A., Sima C.S., Lou F., Dycoco J., Rusch V., Rizk N.P., Jones D.R., Huang J. (2014). Thirty-Day Mortality Underestimates the Risk of Early Death after Major Resections for Thoracic Malignancies. Ann. Thorac. Surg..

[78] Batirel H.F., Metintas M., Caglar H.B., Ak G., Yumuk P.F., Yildizeli B., Yuksel M. (2016). Adoption of Pleurectomy and Decortication for Malignant Mesothelioma Leads to Similar Survival as Extrapleural Pneumonectomy. J. Thorac. Cardiovasc. Surg..

[79] Cho B.C.J., Feld R., Leighl N., Opitz I., Anraku M., Tsao M.-S., Hwang D.M., Hope A., de Perrot M. (2014). A Feasibility Study Evaluating Surgery for Mesothelioma After Radiation Therapy: The “SMART” Approach for Resectable Malignant Pleural Mesothelioma. J. Thorac. Oncol. Off. Publ. Int. Assoc. Study Lung Cancer.

[80] Falanga F., Rinaldi P., Primiceri C., Bortolotto C., Oneta O., Agustoni F., Morbini P., Saracino L., Eleftheriou D., Sottotetti F. (2022). Feasibility and Safety of Extended Pleurectomy/Decortication for Malignant Pleural Mesothelioma. A Single Group Experience. Thorac. cancer.

[81] de Perrot M., Dong Z., Bradbury P., Patsios D., Keshavjee S., Leighl N.B., Hope A., Feld R., Cho J. (2017). Impact of Tumour Thickness on Survival after Radical Radiation and Surgery in Malignant Pleural Mesothelioma. Eur. Respir. J..

[82] van Ruth S., Baas P., Haas R.L.M., Rutgers E.J.T., Verwaal V.J., Zoetmulder F.A.N. (2003). Cytoreductive Surgery Combined with Intraoperative Hyperthermic Intrathoracic Chemotherapy for Stage I Malignant Pleural Mesothelioma. Ann. Surg. Oncol..

[83] Pass H.I., DeLaney T.F., Tochner Z., Smith P.E., Temeck B.K., Pogrebniak H.W., Kranda K.C., Russo A., Friauf W.S., Cole J.W. (1994). Intrapleural Photodynamic Therapy: Results of a Phase I Trial. Ann. Surg. Oncol..

[84] Baas P., Murrer L., Zoetmulder F.A., Stewart F.A., Ris H.B., van Zandwijk N., Peterse J.L., Rutgers E.J. (1997). Photodynamic Therapy as Adjuvant Therapy in Surgically Treated Pleural Malignancies. Br. J. Cancer.

[85] Vogelzang N.J., Rusthoven J.J., Symanowski J., Denham C., Kaukel E., Ruffie P., Gatzemeier U., Boyer M., Emri S., Manegold C. (2003). Phase III Study of Pemetrexed in Combination with Cisplatin versus Cisplatin Alone in Patients with Malignant Pleural Mesothelioma. J. Clin. Oncol. Off. J. Am. Soc. Clin. Oncol..

[86] Zalcman G., Mazieres J., Margery J., Greillier L., Audigier-Valette C., Moro-Sibilot D., Molinier O., Corre R., Monnet I., Gounant V. (2016). Bevacizumab for Newly Diagnosed Pleural Mesothelioma in the Mesothelioma Avastin Cisplatin Pemetrexed Study (MAPS): A Randomised, Controlled, Open-Label, Phase 3 Trial. Lancet.

[87] Tsao A.S., Lindwasser O.W., Adjei A.A., Adusumilli P.S., Beyers M.L., Blumenthal G.M., Bueno R., Burt B.M., Carbone M., Dahlberg S.E. (2018). Current and Future Management of Malignant Mesothelioma: A Consensus Report from the National Cancer Institute Thoracic Malignancy Steering Committee, International Association for the Study of Lung Cancer, and Mesothelioma Applied Research Foundation. J. Thorac. Oncol. Off. Publ. Int. Assoc. Study Lung Cancer.

[88] Bueno R., Stawiski E.W., Goldstein L.D., Durinck S., De Rienzo A., Modrusan Z., Gnad F., Nguyen T.T., Jaiswal B.S., Chirieac L.R. (2016). Comprehensive Genomic Analysis of Malignant Pleural Mesothelioma Identifies Recurrent Mutations, Gene Fusions and Splicing Alterations. Nat. Genet..

[89] Mutti L., Peikert T., Robinson B.W.S., Scherpereel A., Tsao A.S., de Perrot M., Woodard G.A., Jablons D.M., Wiens J., Hirsch F.R. (2018). Scientific Advances and New Frontiers in Mesothelioma Therapeutics. J. Thorac. Oncol. Off. Publ. Int. Assoc. Study Lung Cancer.

[90] Alley E.W., Lopez J., Santoro A., Morosky A., Saraf S., Piperdi B., van Brummelen E. (2017). Clinical Safety and Activity of Pembrolizumab in Patients with Malignant Pleural Mesothelioma (KEYNOTE-028): Preliminary Results from a Non-Randomised, Open-Label, Phase 1b Trial. Lancet. Oncol..

[91] Metaxas Y., Rivalland G., Mauti L.A., Klingbiel D., Kao S., Schmid S., Nowak A.K., Gautschi O., Bartnick T., Hughes B.G. (2018). Pembrolizumab as Palliative Immunotherapy in Malignant Pleural Mesothelioma. J. Thorac. Oncol. Off. Publ. Int. Assoc. Study Lung Cancer.

[92] Nowak A.K., Lesterhuis W.J., Kok P.-S., Brown C., Hughes B.G., Karikios D.J., John T., Kao S.C.-H., Leslie C., Cook A.M. (2020). Durvalumab with First-Line Chemotherapy in Previously Untreated Malignant Pleural Mesothelioma (DREAM): A Multicentre, Single-Arm, Phase 2 Trial with a Safety Run-In. Lancet. Oncol..

[93] Baas P., Scherpereel A., Nowak A.K., Fujimoto N., Peters S., Tsao A.S., Mansfield A.S., Popat S., Jahan T., Antonia S. (2021). First-Line Nivolumab plus Ipilimumab in Unresectable Malignant Pleural Mesothelioma (CheckMate 743): A Multicentre, Randomised, Open-Label, Phase 3 Trial. Lancet.

[94] Fennell D.A., Ewings S., Ottensmeier C., Califano R., Hanna G.G., Hill K., Danson S., Steele N., Nye M., Johnson L. (2021). Nivolumab versus Placebo in Patients with Relapsed Malignant Mesothelioma (CONFIRM): A Multicentre, Double-Blind, Randomised, Phase 3 Trial. Lancet Oncol..

[95] Dozier J., Zheng H., Adusumilli P.S. (2017). Immunotherapy for Malignant Pleural Mesothelioma: Current Status and Future Directions. Transl. Lung Cancer Res..

[96] Kok P.S., Forde P.M., Hughes B., Sun Z., Brown C., Ramalingam S., Cook A., Lesterhuis W.J., Yip S., O’Byrne K. (2022). Protocol of DREAM3R: DuRvalumab with ChEmotherapy as First-Line TreAtment in Advanced Pleural Mesothelioma-a Phase 3 Randomised Trial. BMJ Open.

[97] Belderbos R.A., Baas P., Berardi R., Cornelissen R., Fennell D.A., Van Meerbeeck J.P., Scherpereel A., Vroman H., Aerts J.G.J.V. (2019). A Multicenter, Randomized, Phase II/III Study of Dendritic Cells Loaded with Allogeneic Tumor Cell Lysate (MesoPher) in Subjects with Mesothelioma as Maintenance Therapy after Chemotherapy: DENdritic Cell Immunotherapy for Mesothelioma (DENIM) Trial. Transl. Lung Cancer Res..

[98] Dumoulin D.W., Cornelissen R., Bezemer K., Baart S.J., Aerts J.G.J.V. (2021). Long-Term Follow-up of Mesothelioma Patients Treated with Dendritic Cell Therapy in Three Phase i/Ii Trials. Vaccines.

[99] Field G.C., Pavlyk I., Szlosarek P.W. (2023). Bench-to-Bedside Studies of Arginine Deprivation in Cancer. Molecules.

[100] Chintala N.K., Restle D., Quach H., Saini J., Bellis R., Offin M., Beattie J., Adusumilli P.S. (2021). CAR T-Cell Therapy for Pleural Mesothelioma: Rationale, Preclinical Development, and Clinical Trials. Lung Cancer.

[101] Romero D. (2021). Uncovering Adagrasib Resistance CAR T Cells Show Promise in Mesothelioma. Nat. Rev. Clin. Oncol..

[102] Klampatsa A., Dimou V., Albelda S.M. (2021). Mesothelin-Targeted CAR-T Cell Therapy for Solid Tumors. Expert Opin. Biol. Ther..

[103] Hassan R., Kindler H.L., Jahan T., Bazhenova L., Reck M., Thomas A., Pastan I., Parno J., O’Shannessy D.J., Fatato P. (2014). Phase II Clinical Trial of Amatuximab, a Chimeric Antimesothelin Antibody with Pemetrexed and Cisplatin in Advanced Unresectable Pleural Mesothelioma. Clin. Cancer Res. Off. J. Am. Assoc. Cancer Res..

[104] Golfier S., Kopitz C., Kahnert A., Heisler I., Schatz C.A., Stelte-Ludwig B., Mayer-Bartschmid A., Unterschemmann K., Bruder S., Linden L. (2014). Anetumab Ravtansine: A Novel Mesothelin-Targeting Antibody-Drug Conjugate Cures Tumors with Heterogeneous Target Expression Favored by Bystander Effect. Mol. Cancer Ther..

[105] Hassan R., Blumenschein G.R.J., Moore K.N., Santin A.D., Kindler H.L., Nemunaitis J.J., Seward S.M., Thomas A., Kim S.K., Rajagopalan P. (2020). First-in-Human, Multicenter, Phase I Dose-Escalation and Expansion Study of Anti-Mesothelin Antibody-Drug Conjugate Anetumab Ravtansine in Advanced or Metastatic Solid Tumors. J. Clin. Oncol. Off. J. Am. Soc. Clin. Oncol..

[106] Zhao X.-Y., Subramanyam B., Sarapa N., Golfier S., Dinter H. (2016). Novel Antibody Therapeutics Targeting Mesothelin In Solid Tumors. Clin. Cancer Drugs.

[107] Hollevoet K., Mason-Osann E., Liu X., Imhof-Jung S., Niederfellner G., Pastan I. (2014). In Vitro and in Vivo Activity of the Low-Immunogenic Antimesothelin Immunotoxin RG7787 in Pancreatic Cancer. Mol. Cancer Ther..

[108] Sivick K.E., Desbien A.L., Glickman L.H., Reiner G.L., Corrales L., Surh N.H., Hudson T.E., Vu U.T., Francica B.J., Banda T. (2018). Magnitude of Therapeutic STING Activation Determines CD8(+) T Cell-Mediated Anti-Tumor Immunity. Cell Rep..

[109] Marcus A., Mao A.J., Lensink-Vasan M., Wang L., Vance R.E., Raulet D.H. (2018). Tumor-Derived CGAMP Triggers a STING-Mediated Interferon Response in Non-Tumor Cells to Activate the NK Cell Response. Immunity.

[110] Sen T., Rodriguez B.L., Chen L., Corte C.M., Morikawa N., Fujimoto J., Cristea S., Nguyen T., Diao L., Li L. (2019). Targeting DNA Damage Response Promotes Antitumor Immunity through STING-Mediated T-Cell Activation in Small Cell Lung Cancer. Cancer Discov..

[111] Jenkins R.W., Aref A.R., Lizotte P.H., Ivanova E., Stinson S., Zhou C.W., Bowden M., Deng J., Liu H., Miao D. (2018). Ex Vivo Profiling of PD-1 Blockade Using Organotypic Tumor Spheroids. Cancer Discov..

[112] Voabil P., de Bruijn M., Roelofsen L.M., Hendriks S.H., Brokamp S., van den Braber M., Broeks A., Sanders J., Herzig P., Zippelius A. (2021). An Ex Vivo Tumor Fragment Platform to Dissect Response to PD-1 Blockade in Cancer. Nat. Med..

[113] Ghosh M., Saha S., Bettke J., Nagar R., Parrales A., Iwakuma T., van der Velden A.W.M., Martinez L.A. (2021). Mutant P53 Suppresses Innate Immune Signaling to Promote Tumorigenesis. Cancer Cell.

[114] Lau L., Gray E.E., Brunette R.L., Stetson D.B. (2015). DNA Tumor Virus Oncogenes Antagonize the CGAS-STING DNA-Sensing Pathway. Science.

[115] Knelson E.H., Ivanova E.V., Tarannum M., Campisi M., Lizotte P.H., Booker M.A., Ozgenc I., Noureddine M., Meisenheimer B., Chen M. (2022). Activation of Tumor-Cell STING Primes NK-Cell Therapy. Cancer Immunol. Res..

[116] Theelen W.S.M.E., Peulen H.M.U., Lalezari F., van der Noort V., de Vries J.F., Aerts J.G.J.V., Dumoulin D.W., Bahce I., Niemeijer A.-L.N., de Langen A.J. (2019). Effect of Pembrolizumab After Stereotactic Body Radiotherapy vs Pembrolizumab Alone on Tumor Response in Patients With Advanced Non-Small Cell Lung Cancer: Results of the PEMBRO-RT Phase 2 Randomized Clinical Trial. JAMA Oncol..

[117] Xu N., Palmer D.C., Robeson A.C., Shou P., Bommiasamy H., Laurie S.J., Willis C., Dotti G., Vincent B.G., Restifo N.P. (2021). STING Agonist Promotes CAR T Cell Trafficking and Persistence in Breast Cancer. J. Exp. Med..

[118] Zhang J., Li Y., Zou J., Lai C.-T., Zeng T., Peng J., Zou W., Cao B., Liu D., Zhu L.-Y. (2022). Comprehensive Analysis of the Glutathione S-Transferase Mu (GSTM) Gene Family in Ovarian Cancer Identifies Prognostic and Expression Significance. Front. Oncol..

[119] Cerne D., Melkic E., Trost Z., Sok M., Marc J. (2007). Lipoprotein Lipase Activity and Gene Expression in Lung Cancer and in Adjacent Noncancer Lung Tissue. Exp. Lung Res..

[120] Trempolec N., Degavre C., Doix B., Brusa D., Corbet C., Feron O. (2020). Acidosis-Induced TGF-Β2 Production Promotes Lipid Droplet Formation in Dendritic Cells and Alters Their Potential to Support Anti-Mesothelioma T Cell Response. Cancers.

[121] Englinger B., Laemmerer A., Moser P., Kallus S., Röhrl C., Pirker C., Baier D., Mohr T., Niederstaetter L., Meier-Menches S.M. (2020). Lipid Droplet-Mediated Scavenging as Novel Intrinsic and Adaptive Resistance Factor against the Multikinase Inhibitor Ponatinib. Int. J. Cancer.

[122] Yan H.H., Pickup M., Pang Y., Gorska A.E., Li Z., Chytil A., Geng Y., Gray J.W., Moses H.L., Yang L. (2010). Gr-1+CD11b+ Myeloid Cells Tip the Balance of Immune Protection to Tumor Promotion in the Premetastatic Lung. Cancer Res..

[123] Wang Y., Li M., Zhang L., Chen Y., Ha M. (2022). LINC01140 Inhibits Nonsmall Cell Lung Cancer Progression and Cisplatin Resistance through the MiR-4742-5p/TACC1 Axis. J. Biochem. Mol. Toxicol..

[124] Hmeljak J., Sanchez-Vega F., Hoadley K.A., Shih J., Stewart C., Heiman D., Tarpey P., Danilova L., Drill E., Gibb E.A. (2018). Integrative Molecular Characterization of Malignant Pleural Mesothelioma. Cancer Discov..

[125] Winkler J., Abisoye-Ogunniyan A., Metcalf K.J., Werb Z. (2020). Concepts of Extracellular Matrix Remodelling in Tumour Progression and Metastasis. Nat. Commun..

[126] Bonnans C., Chou J., Werb Z. (2014). Remodelling the Extracellular Matrix in Development and Disease. Nat. Rev. Mol. Cell Biol..

[127] Chew S.H., Toyokuni S. (2015). Malignant Mesothelioma as an Oxidative Stress-Induced Cancer: An Update. Free Radic. Biol. Med..

[128] Chaudhuri O., Koshy S.T., Branco da Cunha C., Shin J.-W., Verbeke C.S., Allison K.H., Mooney D.J. (2014). Extracellular Matrix Stiffness and Composition Jointly Regulate the Induction of Malignant Phenotypes in Mammary Epithelium. Nat. Mater..

[129] Chakravarthy A., Khan L., Bensler N.P., Bose P., De Carvalho D.D. (2018). TGF-β-Associated Extracellular Matrix Genes Link Cancer-Associated Fibroblasts to Immune Evasion and Immunotherapy Failure. Nat. Commun..

[130] Geiger R., Rieckmann J.C., Wolf T., Basso C., Feng Y., Fuhrer T., Kogadeeva M., Picotti P., Meissner F., Mann M. (2016). L-Arginine Modulates T Cell Metabolism and Enhances Survival and Anti-Tumor Activity. Cell.

[131] Ollila H., Mäyränpää M.I., Paavolainen L., Paajanen J., Välimäki K., Sutinen E., Wolff H., Räsänen J., Kallioniemi O., Myllärniemi M. (2022). Prognostic Role of Tumor Immune Microenvironment in Pleural Epithelioid Mesothelioma. Front. Oncol..

[132] Creaney J., Patch A.-M., Addala V., Sneddon S.A., Nones K., Dick I.M., Lee Y.C.G., Newell F., Rouse E.J., Naeini M.M. (2022). Comprehensive Genomic and Tumour Immune Profiling Reveals Potential Therapeutic Targets in Malignant Pleural Mesothelioma. Genome Med..

[133] Lievense L.A., Cornelissen R., Bezemer K., Kaijen-Lambers M.E.H., Hegmans J.P.J.J., Aerts J.G.J. (2016). V Pleural Effusion of Patients with Malignant Mesothelioma Induces Macrophage-Mediated T Cell Suppression. J. Thorac. Oncol. Off. Publ. Int. Assoc. Study Lung Cancer.

[134] Magkouta S.F., Vaitsi P.C., Pappas A.G., Iliopoulou M., Kosti C.N., Psarra K., Kalomenidis I.T. (2021). CSF1/CSF1R Axis Blockade Limits Mesothelioma and Enhances Efficiency of Anti-PDL1 Immunotherapy. Cancers.

[135] Torricelli F., Donati B., Reggiani F., Manicardi V., Piana S., Valli R., Lococo F., Ciarrocchi A. (2023). Spatially Resolved, High-Dimensional Transcriptomics Sorts out the Evolution of Biphasic Malignant Pleural Mesothelioma: New Paradigms for Immunotherapy. Mol. Cancer.

[136] Panou V., Gadiraju M., Wolin A., Weipert C.M., Skarda E., Husain A.N., Patel J.D., Rose B., Zhang S.R., Weatherly M. (2018). Frequency of Germline Mutations in Cancer Susceptibility Genes in Malignant Mesothelioma. J. Clin. Oncol. Off. J. Am. Soc. Clin. Oncol..

[137] Guo G., Chmielecki J., Goparaju C., Heguy A., Dolgalev I., Carbone M., Seepo S., Meyerson M., Pass H.I. (2015). Whole-Exome Sequencing Reveals Frequent Genetic Alterations in BAP1, NF2, CDKN2A, and CUL1 in Malignant Pleural Mesothelioma. Cancer Res..

[138] Schelch K., Emminger D., Zitta B., Johnson T.G., Kopatz V., Eder S., Ries A., Stefanelli A., Heffeter P., Hoda M.A. (2023). Targeting YB-1 via Entinostat Enhances Cisplatin Sensitivity of Pleural Mesothelioma in Vitro and in Vivo. Cancer Lett..

[139] Bott M., Brevet M., Taylor B.S., Shimizu S., Ito T., Wang L., Creaney J., Lake R.A., Zakowski M.F., Reva B. (2011). The Nuclear Deubiquitinase BAP1 Is Commonly Inactivated by Somatic Mutations and 3p21.1 Losses in Malignant Pleural Mesothelioma. Nat. Genet..

[140] Srinivasan G., Sidhu G.S., Williamson E.A., Jaiswal A.S., Najmunnisa N., Wilcoxen K., Jones D., George T.J.J., Hromas R. (2017). Synthetic Lethality in Malignant Pleural Mesothelioma with PARP1 Inhibition. Cancer Chemother. Pharmacol..

[141] Passiglia F., Bironzo P., Righi L., Listì A., Arizio F., Novello S., Volante M., Scagliotti G.V. (2021). A Prospective Phase II Single-Arm Study of Niraparib Plus Dostarlimab in Patients With Advanced Non-Small-Cell Lung Cancer and/or Malignant Pleural Mesothelioma, Positive for PD-L1 Expression and Germline or Somatic Mutations in the DNA Repair Genes. Rat. Clin. Lung Cancer.

[142] Fennell D.A., King A., Mohammed S., Branson A., Brookes C., Darlison L., Dawson A.G., Gaba A., Hutka M., Morgan B. (2021). Rucaparib in Patients with BAP1-Deficient or BRCA1-Deficient Mesothelioma (MiST1): An Open-Label, Single-Arm, Phase 2a Clinical Trial. Lancet. Respir. Med..

[143] Parisi A., Rossi F., De Filippis C., Paoloni F., Felicetti C., Mammarella A., Pecci F., Lupi A., Berardi R. (2023). Current Evidence and Future Perspectives about the Role of PARP Inhibitors in the Treatment of Thoracic Cancers. Onco. Targets. Ther..

[144] van Zandwijk N., Pavlakis N., Kao S.C., Linton A., Boyer M.J., Clarke S., Huynh Y., Chrzanowska A., Fulham M.J., Bailey D.L. (2017). Safety and Activity of MicroRNA-Loaded Minicells in Patients with Recurrent Malignant Pleural Mesothelioma: A First-in-Man, Phase 1, Open-Label, Dose-Escalation Study. Lancet. Oncol..

[145] Witta S.E., Jotte R.M., Konduri K., Neubauer M.A., Spira A.I., Ruxer R.L., Varella-Garcia M., Bunn P.A.J., Hirsch F.R. (2012). Randomized Phase II Trial of Erlotinib with and without Entinostat in Patients with Advanced Non-Small-Cell Lung Cancer Who Progressed on Prior Chemotherapy. J. Clin. Oncol. Off. J. Am. Soc. Clin. Oncol..

[146] Juergens R.A., Wrangle J., Vendetti F.P., Murphy S.C., Zhao M., Coleman B., Sebree R., Rodgers K., Hooker C.M., Franco N. (2011). Combination Epigenetic Therapy Has Efficacy in Patients with Refractory Advanced Non-Small Cell Lung Cancer. Cancer Discov..

[147] Maiti A., Qi Q., Peng X., Yan L., Takabe K., Hait N.C. (2019). Class I Histone Deacetylase Inhibitor Suppresses Vasculogenic Mimicry by Enhancing the Expression of Tumor Suppressor and Anti-Angiogenesis Genes in Aggressive Human TNBC Cells. Int. J. Oncol..

[148] El-Naggar A.M., Somasekharan S.P., Wang Y., Cheng H., Negri G.L., Pan M., Wang X.Q., Delaidelli A., Rafn B., Cran J. (2019). Class I HDAC Inhibitors Enhance YB-1 Acetylation and Oxidative Stress to Block Sarcoma Metastasis. EMBO Rep..

[149] Sidiropoulos D.N., Rafie C.I., Jang J.K., Castanon S., Baugh A.G., Gonzalez E., Christmas B.J., Narumi V.H., Davis-Marcisak E.F., Sharma G. (2022). Entinostat Decreases Immune Suppression to Promote Antitumor Responses in a HER2+ Breast Tumor Microenvironment. Cancer Immunol. Res..

[150] Truong A.S., Zhou M., Krishnan B., Utsumi T., Manocha U., Stewart K.G., Beck W., Rose T.L., Milowsky M.I., He X. (2021). Entinostat Induces Antitumor Immune Responses through Immune Editing of Tumor Neoantigens. J. Clin. Investig..

